# Card9 protects sepsis by regulating Ripk2-mediated activation of NLRP3 inflammasome in macrophages

**DOI:** 10.1038/s41419-022-04938-y

**Published:** 2022-05-26

**Authors:** Zhen Xu, Daoqian Li, Wei Qu, Yuxin Yin, Shuping Qiao, Yanan Zhu, Sunan Shen, Yayi Hou, Jie Yang, Tingting Wang

**Affiliations:** 1 The State Key Laboratory of Pharmaceutical Biotechnology, Division of Immunology, Medical School, Nanjing University, Nanjing, 210093 China; 2 Jiangsu Key Laboratory of Molecular Medicine, Division of Immunology, Medical School, Nanjing University, Nanjing, 210093 China; 3 Nanjing Stomatological Hospital, Medical School of Nanjing University, Nanjing, 210093 China; 4 College of life sciences, Nanjing University, Nanjing, 210023 China

**Keywords:** Immunology, Molecular biology

## Abstract

Sepsis is characterized by systemic inflammation, it’s caused by primary infection of pathogenic microorganisms or secondary infection of damaged tissue. In this study, we focus on sepsis-induced intestine barrier functional disturbalice, presenting as increased permeability of intestinal epithelium. We observed that the phenotype of LPS-induced sepsis was exacerbated in *Card9*^*−/−*^ mice, especially displaying more serious intestinal inflammation and gut barrier dysfunction. Next, we found the hyperactivation of NLRP3 inflammasome in the intestinal macrophages of *Card9*^*−/−*^-sepsis mice. Moreover, Card9 over-expression decreased NLRP3 inflammasome activation in macrophages. Furthermore, we found that Card9 inhibited NLRP3 inflammasome activation by recruiting Ripk2. The competitive binding between Ripk2 with Caspase-1, instead of ASC with Caspase-1, inhibited the NLRP3 inflammasome activation. Over-expression of Ripk2 alleviated septic intestinal injury caused by Card9 deficiency. Taken together, we suggested Card9 acts as a negative regulation factor of NLRP3 inflammasome activation, which protects against intestinal damage during sepsis. Therefore, maintaining Card9-Ripk2 signaling homeostasis may provide a novel therapy of septic intestinal damage.

## Introduction

Recently, sepsis was redefined as a life-threatening multiorgan dysfunction syndrome because of the maladjusted inflammatory response to infection [1, 2]. During sepsis, the intestinal microenvironment is disrupted, leading to pathologic changes that cause both local and distant damage [3–5]. Numerous studies have shown that intestinal integrity is impaired, cell apoptosis is increased and intestinal barrier permeability is changed in sepsis patients, because of increased production of pro-inflammatory cytokines and interferon [6, 7]. Destruction of the intestinal barrier can lead to fatal sepsis and multiple organ failure [8].

In the early phase of sepsis, there is a violent storm of inflammation. The deterioration of any inflammatory reactions can endanger the lives of patients with sepsis [2, 9–11]. Inflammation is usually caused by induction of pathogen-related or damage-related molecular patterns [12, 13]. NLRP3 Inflammasome is an important signal complex formed by the subset of pattern-recognition receptors. Priming is the step one of NLRP3 activation, referred to Signal 1, which induces the transcription of pro-IL-1β and NLRP3 [14]. Compared to Signal 1, NLRP3 inflammasome activation (Signal 2) includes all events participated in the complex formation and the activation of caspase-1. Upon stimulation, NLRP3 binds with ASC (apoptosis associated speck-like protein containing CARD) though its pyrin domain. ASC recruits the effector pro-Caspase-1 though CARD-CARD interaction to form a cytoplasmic complex [15]. Assembly of inflammasome induces the cleavage and activation of caspase-1, transforming pro-IL-1β and pro-IL-18 into their mature forms [16–18]. Previous research manifests that NLRP3 inflammasome components are overexpressed in the white blood corpuscles of sepsis patients [19]. The original purpose of inflammasome activation is to protect the host from pathogens, but the potent proinflammatory activities of IL-1β requires that the activity of inflammasome should be strictly controlled [20, 21].

The adaptor protein Card9 is restricted expression in myeloid cells [22, 23]. Card9 was defined as the negative regulatory in production of IL-1β under bacterial infections, which is in sharp contrast to its role in driving pro-inflammatory responses in fungal infections [24]. Previous study already showed that Card9 regulates expression of IL-1β through NF-κB [25, 26], which mediated transcription of IL-1β. In our study, we established LPS-induced sepsis model in wild-type (WT) and *Card9*^*−/−*^ mice. We observed that *Card9*^*−/−*^ mice are susceptible to sepsis, accompanied by significant intestinal inflammation and intestinal barrier dysfunction. We also confirmed that Card9 regulated the activation of NLRP3 inflammasome in macrophages in a Ripk2-dependent manner.

## Result

### *Card9*^*−/−*^ mice are susceptible to sepsis and along with intestinal inflammation and gut barrier dysfunction

**Fig. 1:**
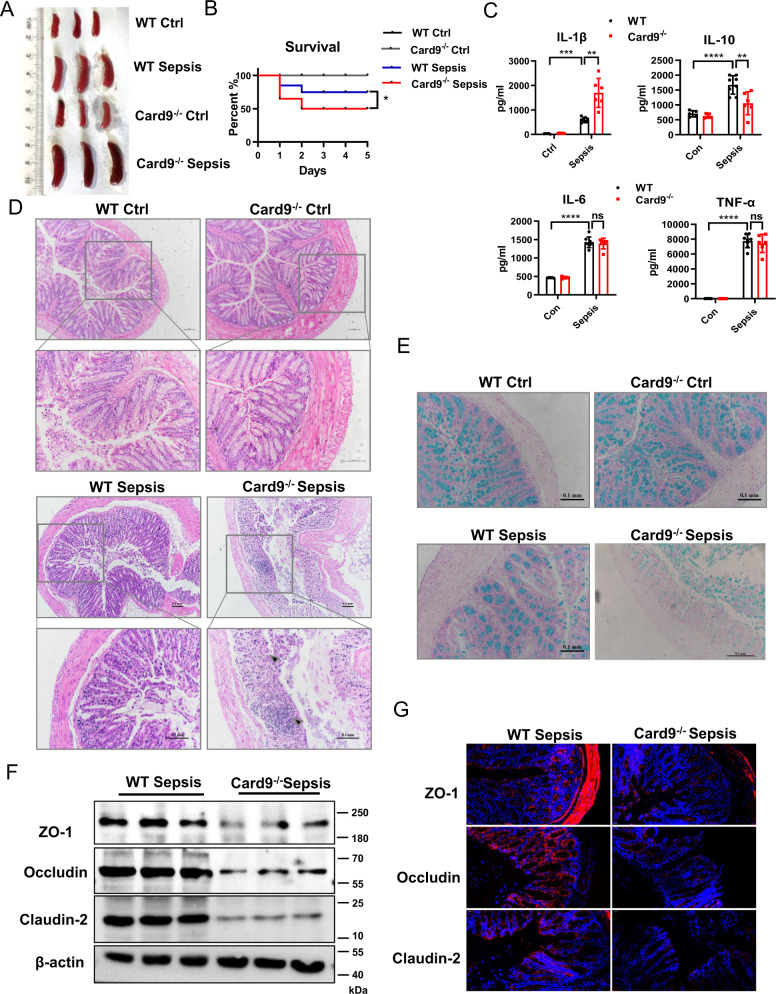
*Card9*^*−/−*^ mice are susceptible to sepsis and along with intestinal inflammation and gut barrier dysfunction. WT mice and *Card9*^*−/−*^ littermates (*n* = 10 for each group) were intraperitoneal injection with LPS (5 mg/kg). **A** Spleen photo of mice in control and experimental groups. **B** Survival rate of WT mice and *Card9*^*−/−*^ mice within 5 days after LPS treatment. **C** Secretion of IL-1β, IL-10, IL-6 and TNF-α in serum was assessed by ELISA. **D** Histological analysis of intestinal was assessed by H&E staining. **E** Intestinal tissue from WT mice and *Card9*^*−/−*^ mice were staining with alcian blue. **F** Western blot analysis of ZO-1, Occludin and Claudin-2 in intestinal tissue from WT-sepsis mice and *Card9*^*−/−*^-sepsis mice. **G** Immunofluorescence analysis of ZO-1, Occludin and Claudin-2 in the intestinal mucosa. Data are shown as mean ± SEM. Scale bars, 0.1 mm. Each group was representative of at least three biological replicates. *p < 0.05, **p < 0.01, ***p < 0.001.

To evaluate the function of Card9 in sepsis, we established LPS-induced sepsis in WT and *Card9*^*−/−*^ mice. Mice were sacrificed 24 h after LPS injection. We found the spleen is visibly enlarged in the *Card9*^*−/−*^ mice compared with WT mice after LPS administration (Fig. 1A and Fig. S1A). Meanwhile, the survival rate was significantly lower of *Card9*^*−/−*^-sepsis mice than that of WT-sepsis mice (Fig. 1B). Since sepsis is a systemic inflammation and can cause various organ damage, we detected the expression of aspartate aminotransferase (AST), alanine aminotransferase (ALT), creatinine and amylase (AMY) in serum. Increased expressions of AST and ALT, representing worsening liver damage were noted in *Card9*^*−/−*^-sepsis mice compared with WT-sepsis mice. However, there were no remarkable differences in creatinine and AMY protein levels between *Card9*^*−/−*^-sepsis and WT-sepsis mice (Fig. S1B). Furthermore, upon LPS treatment, *Card9*^*−/−*^ mice had higher level of IL-1β and lower level of IL-10 in serum, when compared with WT mice. But similar concentration of TNF-α and IL-6 was observed between WT-sepsis and *Card9*^*−/−*^-sepsis mice (Fig. 1C). Sepsis is characterized by multiple organ damage, we performed histological evaluation of tissue injury. More inflammatory cells infiltration and increased destruction of intestinal mucosa structure were observed in *Card9*^*−/−*^-sepsis mice compared with WT-sepsis mice (Fig. 1D). By using alcian blue staining, the damage of intestinal barrier was obviously aggravated in *Card9*^*−/−*^-sepsis mice, compared with WT-sepsis mice (Fig. 1E). Moreover, we detected substantially decreased expression of tight junction proteins in *Card9*^*−/−*^-sepsis mice compared with WT-sepsis mice, including ZO-1, occludin and Claudin-2 (Fig. 1F, G). Meanwhile, histological evaluation of liver and lung manifested there were no differences between WT-sepsis and *Card9*^*−/−*^-sepsis mice (Fig. S1C, D). These data suggested that *Card9*^*−/−*^ mice are more susceptible to sepsis than WT mice, presenting as more serious damage to intestinal function in *Card9*^*−/−*^ mice.

### Excessive activation of NLRP3 inflammasome by sepsis in *Card9*^*−/−*^ mice intestinal mucosa

**Fig. 2:**
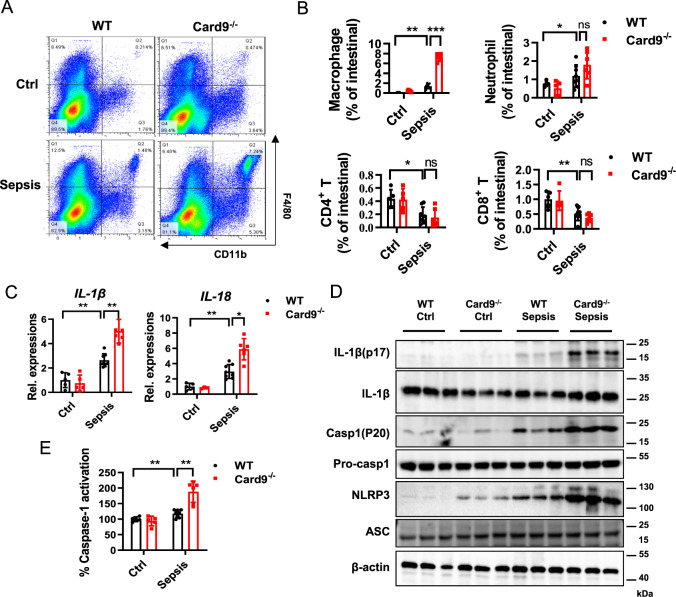
Excessive activation of NLRP3 inflammasome in *Card9*^*−/−*^ mice. **A** The percentage of macrophages (F4/80^+^ and CD11b^+^ cells) in intestinal cells from WT mice and *Card9*^*−/−*^ mice was detected by flow cytometry. **B** The percentage of macrophages, neutrophil, CD4^+^T cells (CD3^+^ and CD4^+^) and CD8^+^T cells (CD3^+^ and CD8^+^) in intestinal cells from WT mice and *Card9*^*−/−*^ mice was quantified. **C**–**E** Macrophages (F4/80^+^ cells) were isolated from intestinal tissue of WT mice and *Card9*^*−/−*^ mice. **C** mRNA expression of *IL-1β* and *IL-18* in macrophages detected by RT-PCR. **D** Western blot analysis of NLRP3 inflammasome associated proteins. **E** The percentage of Caspase-1 activation was detected by Caspase-1 Fluorometric Assay Kit. Data are shown as mean ± SEM. Each group was representative of at least three biological replicates. *p < 0.05, **p < 0.01, ***p < 0.001.

To explore the mechanism of intestinal injury caused by the loss of Card9, we tested the changes of immune cells in the intestine. As shown in Fig. 2A, the number of infiltrating mononuclear macrophages (F4/80^+^CD11b^+^cells) in the intestinal cells was increased in *Card9*^*−/−*^-sepsis mice compared with WT-sepsis mice. The proportion of neutrophil (CD11b^+^Ly6G^+^) and the adaptive T cells (CD4^+^T cells and CD8^+^T cells) in intestinal cells showed no difference between WT-sepsis mice and *Card9*^*−/−*^-sepsis mice (Fig. 2B). Next, we isolated primary macrophages (F4/80^+^ cells) from intestine cells using MACS. We found an obviously higher transcriptional levels of IL-1β and IL-18 in macrophages in *Card9*^*−/−*^-sepsis mice (Fig. 2C). In addition, excessive expression of NLRP3 were more obvious in macrophages from *Card9*^*−/−*^-sepsis mice compared with WT-sepsis mice (Fig. 2D). To determine whether Card9 has the regulation in other inflammasomes, we detected the expression of NLRC4 and AIM2 in intestinal macrophages. The results showed that the expression of NLRC4 and AIM2 had no difference between WT-sepsis and *Card9*^*−/−*^-sepsis mice (Fig. S1E). Here, we provided the evidence that Card9 has a role in the transcriptional levels of the NLRP3 inflammasome-associated proteins. We also observed that the expression of IL-1β (p17) and Caspase (p20) was increased in macrophages from *Card9*^*−/−*^-sepsis mice compared with WT-sepsis mice (Fig. 2D). Consistently, Caspase-1 activation was more evident in macrophages from *Card9*^*−/−*^-sepsis mice compared with those from WT-sepsis mice (Fig. 2E). These findings demonstrate that Card9 inhibits NLRP3 inflammasome activation in vivo, and its deficiency leads to overactivation of NLRP3 inflammasome in the context of inflammatory diseases. Previous studies have been proved that Card9 regulates “signal 1” of NLRP3 inflammasome through NF-κB [25, 26]. Based on our observations in vivo, we wondered whether Card9 had other direct regulatory role in “signal 2” of NLRP3 inflammasome activation.

### Card9 inhibits NLRP3 inflammasome activation in macrophage

**Fig. 3:**
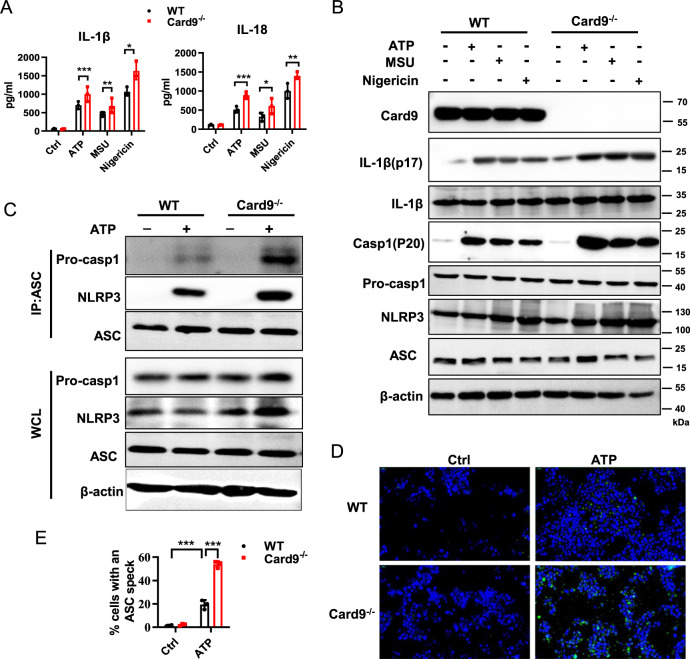
Card9 deficiency results in excessive activation of NLRP3 inflammasome in vitro. **A**, **B** BMDMs were obtained from WT mice and *Card9*^*−/−*^ mice. Cells were unstimulated (medium) or pretreatment with LPS (100 ng/ml) for 3 h, and then respectively treated with ATP (5 mM, 1 h), MSU (500 µg/ml, 2 h) or Nigericin (10 µM, 2 h). **A** ELISA analysis of IL-1β and IL-18 secretion in the culture supernatant. **B** Western blot analysis of cell lysates. **C**–**E** BMDMs were obtained from WT mice and *Card9*^*−/−*^ mice, cells were unstimulated (medium) or were primed with 100 ng/ml LPS for 3 h, and then treated with ATP (5 mM, 1 h). **C** Lysates of BMDMs immunoprecipitated with anti-ASC and then conducted immunoblot analysis. **D, E** Representative immunofluorescence images of ASC speck formation in BMDMs and quantification. Data are shown as mean ± SEM. Scale bars, 0.2 μm. Each group was representative of at least three biological replicates. *p < 0.05, **p < 0.01, ***p < 0.001.

It has been reported that the activation of inflammasome leads to excessive inflammation and tissue damage. We want to know if Card9 is involved in NLRP3 activation. Bone marrow-derived macrophages (BMDMs) were obtained from WT and *Card9*^*−/−*^ mice. After stimulating with ATP, MSU or Nigericin, we observed increased production of IL-1β and IL-18 and increased expression of IL-1β (p17) and caspase-1 (p20) in BMDMs isolated from *Card9*^*−/−*^ mice, compared with that from WT mice (Fig. 3A, B). The assembly of NLRP3/ASC/pro-caspase-1 complex, which is a characteristic of Signal 2, was also enhanced upon ATP treatment when Card9 is defective (Fig. 3C). The activation of NLRP3 inflammasome induces the ASC oligomerization, resulting in ASC specks formation [27, 28]. Accordingly, the deletion of Card9 markedly inhibited the formation of ASC specks after ATP treatment.

**Fig. 4:**
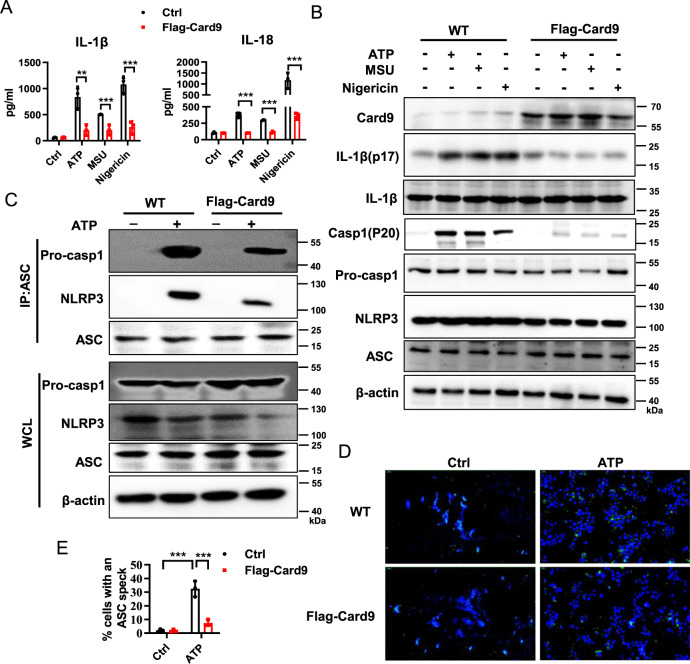
Card9 over-expression decreases LPS-induced NLRP3 inflammasome activation in vitro. **A**, **B** BMDMs were transfected with Card9 over-expression plasmid. Cells were unstimulated (medium) or pretreatment with LPS (100 ng/ml) for 3 h, and then respectively treated with ATP (5 mM, 1 h), MSU (500 µg/ml, 2 h) or Nigericin (10 µM, 2 h). **A** ELISA analysis of IL-1β and IL-18 secretion in the culture supernatant. **B** Western blot analysis of BMDMs lysates. **C**–**E** BMDMs were transfected with Card9 over-expression plasmid, cells were unstimulated (medium) or pretreatment with LPS (100 ng/ml) for 3 h, and then stimulated with ATP (5 mM, 1 h). **C** Lysates of BMDMs immunoprecipitated with anti-ASC and then conducted immunoblot analysis. **D**, **E** Representative immunofluorescence images of ASC speck formation in BMDMs and quantification. Data are shown as mean ± SEM. Scale bars, 0.2μm. Each group was representative of at least three biological replicates. **p < 0.01, ***p < 0.001.

Moreover, BMDMs were transfected with overexpression plasmid Flag-Card9 and showed suppressed expression of IL-1β, IL-18, IL-1β (p17) and caspase-1 (p20) after ATP, MSU or Nigericin treatment (Fig. 4A, B). Similarly, after over-expression of Card9, the assembly of NLRP3/ASC/pro-caspase-1 complex and the formation of ASC specks were decreased after ATP treatment (Fig. 4C–E). Collectively, the observations indicate that Card9 is a negative regulator of NLRP3 inflammasome activation in macrophages.

To address whether Card9 has a broader role in inflammasome activation beyond NLRP3, WT and Card9-deficient macrophages were transfected with poly (dA: dT) to activate AIM2 or treated with NLRC4 activator, Flagellin. IL-1β production and caspase-1 activation showed no difference in macrophages from WT or Card9-deficient mice after poly (dA: dT) transfection or Flagellin treatment (Fig. S2A,B). In addition, we detected AIM2 and NLRC4 inflammasome activation in WT and Flag-Card9 macrophages (Fig. S2C,D). The result also showed that Card9 was not involved in the activation of AIM2 and NLRC4 inflammasome. These data suggest that Card9 is dispensable for AIM2 and NLRC4 inflammasome activation.

These in vivo and in vitro data suggested that the function of Card9 in NLRP3 inflammasome regulation was cofounded by regulating transcriptional levels of the NLRP3 inflammasome-associated proteins and inhibiting NLRP3 inflammasome activation. But, the regulatory mechanism of Card9 regulates NLRP3 inflammasome activation is unclear.

### Card9 inhibits NLRP3 inflammasome activation in a Ripk2-dependent manner

**Fig. 5:**
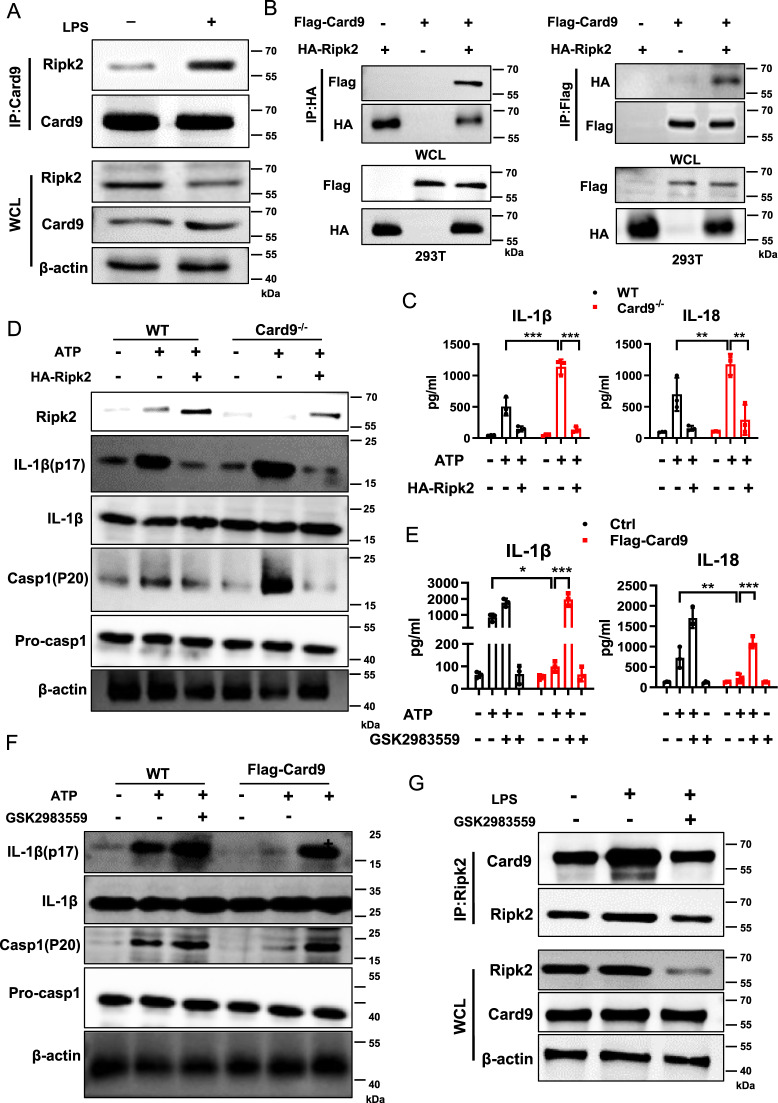
Card9 inhibits NLRP3 inflammasome activation in a Ripk2-dependent manner. **A** Western blot analysis of interaction of Card9 and Ripk2 in BMDMs treated with LPS using co-immunoprecipitation (Co-IP). **B** Western blot of reciprocal Co-IP from HEK293T cells transfecting with Flag-tagged Card9 and HA-tagged Ripk2. **C, D** BMDMs were obtained from WT mice and *Card9*^*−/−*^ mice and transfected with Ripk2 over-expression plasmid, cells were unstimulated (medium) or pretreatment with LPS (100 ng/ml) for 3 h, and then treated with ATP (5 mM, 1 h). **C** ELISA analysis of IL-1β and IL-18 secretion in the culture supernatant. **D** Western blot analysis of cell lysates. **E**, **F** BMDMs were transfected with Card9 over-expression plasmid, cells untreated or treated with Ripk2 inhibitor GSK2983559 (10 μM) for 1 h, followed by unstimulated (medium) or LPS + ATP treatment. **E** ELISA analysis of IL-1β and IL-18 secretion in the culture supernatant. **F** Western blot analysis of cell lysates. **G** Immunoblot analysis of Co-IP from BMDMs untreated or treated with Ripk2 inhibitor GSK2983559 (10 μM) for 1 h, followed by LPS treatment. Data are shown as mean ± SEM. Each group was representative of at least three biological replicates. *p < 0.05, **p < 0.01, ***p < 0.001.

Next, we aimed to reveal the mechanism of Card9 regulates “signal 2” of NLRP3 activation. Functional correlation network analysis shows that the main interaction part of Card9 is composed of key immune and death signaling pathways (Fig. S3A). We examined Card9-interacting proteins using Co-immunoprecipitation assay. The result showed that the interaction between Card9 and Ripk2 was remarkably intensified in macrophages after LPS stimulation (Fig. 5A and Fig. S3B). This finding prompted us to further study whether Card9 actually combined with Ripk2 in NLRP3 inflammasome activation. Co-immunoprecipitation assay showed that Card9 interacted with Ripk2 in HEK293T cells (Fig. 5B). We also over-expressed Ripk2 in BMDMs from WT and *Card9*^*−/−*^ mice. Overexpression of Ripk2 inhibited ATP-induced NLRP3 activation, presenting as decreased production of IL-1β, IL-18 and levels of IL-1β (p17) and caspase-1 (p20) (Fig. 5C, D). Consistently, the attenuated activation of NLRP3 inflammasome caused by overexpression of Card9 was reversed by Ripk2 inhibitor GSK2983559, resulting in enhanced NLRP3 inflammasome activation after ATP treatment (Fig. 5E, F). Furthermore, Ripk2 inhibitor GSK2983559 interrupted the interaction of Card9 and Ripk2 (Fig. 5G). Collectively, these results demonstrate that Card9 works as a negative regulator of NLRP3 inflammasome activation by recruiting Ripk2.

### Competition between Ripk2 and ASC for Caspase-1 interaction

**Fig. 6:**
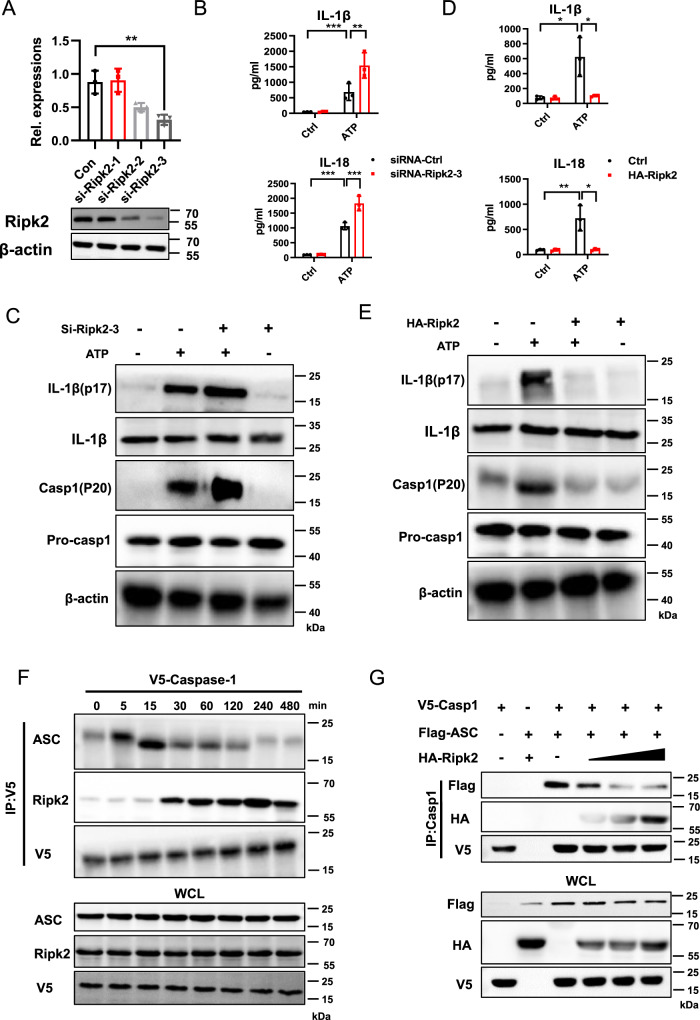
Competition between Ripk2 and ASC for Caspase-1 interaction. **A** BMDMs were transfected with si-Ctrl or si-Ripk2, mRNA expression was detected using qPCR and protein level was detected using western blotting. **B**, **C** BMDMs were transfected with siRNA-Ctrl or siRNA-Ripk2-3. cells were unstimulated (medium) or pretreatment with LPS (100 ng/ml) for 3 h, and then treated with ATP (5 mM, 1 h). **B** ELISA analysis of IL-1β and IL-18 secretion in the culture supernatant. **C** Western blot analysis of cell lysates of BMDMs. **D, E** BMDMs were transfected with Ripk2 over-expression plasmid, cells were unstimulated (medium) or pretreatment with LPS (100 ng/ml) for 3 h, and then treated with ATP (5 mM, 1 h). **D** ELISA analysis of IL-1β and IL-18 secretion in the culture supernatant. **E** Western blot analysis of cell lysates of BMDMs. **F** Differential Caspase-1 interactions upon LPS + ATP stimulation. BMDMs containing V5-Caspase-1 were stimulated with LPS + ATP for the indicated times, followed by IP with anti-V5 and IB with anti-ASC or anti-Ripk2. **G** Competition between ASC and Ripk2 for Caspase-1 interaction. HEK293T cells post-transfection with V5-Caspase-1 or Flag-ASC together with increasing amounts of HA-Ripk2 for 48 h. Then, anti-V5 was used for IP and anti-Flag, anti-HA, or anti-V5 were used for IB. Data are shown as mean ± SEM. Each group was representative of at least three biological replicates. *p < 0.05, **p < 0.01, ***p < 0.001.

The interaction of Card9 and Ripk2 prompted us to confirm the role of Ripk2 in NLRP3 inflammasome activation. The expression of Ripk2 was inhibited in macrophages after transfecting of Ripk2 siRNA (Fig. 6A). Compared with control group, we found the secretions of IL-1β and IL-18, and the expression of IL-1β (p17) and caspase-1 (p20) were significantly increased after inhibiting Ripk2 upon ATP stimulated (Fig. 6B, C). Consistently, overexpression of Ripk2 markedly attenuated ATP-induced NLRP3 inflammasome activation (Fig. 6D, E). To confirm the function of Ripk2 in NLRP3 inflammasome activation, HEK293T cells were overexpressed with Caspase-1. Unlike the Caspase-1/ASC interaction, the interaction between Caspase-1/Ripk2 was initially weak, but increased significantly after 120 min of ATP stimulation (Fig. 6F). Moreover, Ripk2 overexpression disrupted the interaction between Caspase-1 and ASC in a dose-dependent manner (Fig. 6G). These data indicate that Ripk2, competitively binding with Caspase-1, regulated NLRP3 inflammasome activation in macrophages.

### Over-expression of Ripk2 alleviates intestinal damage in Card9^-/-^ sepsis mice

**Fig. 7:**
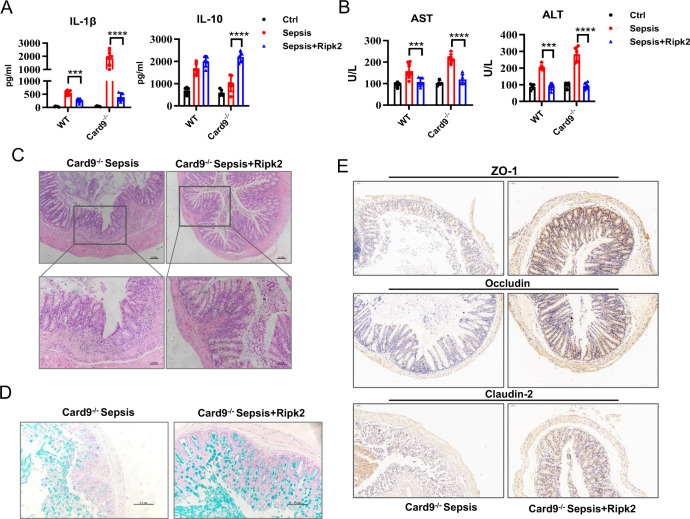
Ripk2 over-expression alleviates intestinal damage caused by Card9 deficiency in sepsis. WT mice and *Card9*^*−/−*^ mice (*n* = 10 for each group) were treated with LPS after 24 h Ad-Ripk2 injected into tail vein. **A** Secretion of IL-1β and IL-10 in serum was assessed by ELISA. **B** Production of AST and ALT was detected in serum. **C** Histological analysis of intestinal was shown by H&E staining. **D** Intestinal tissue from *Card9*^*−/−*^ mice was staining with alcian blue. **E** Immumohistochemical analysis of ZO-1, Occludin and Claudin-2 in intestinal tissue from *Card9*^*−/−*^ mice. Data are shown as mean ± SEM. Each group was representative of at least three biological replicates. **p < 0.01, ***p < 0.001.

The above data proved that Card9 recruits Ripk2 and participates in NLRP3 inflammasome activation in vitro. The function of Ripk2 was further demonstrated in WT and *Card9*^*−/−*^-sepsis mice. WT mice and *Card9*^*−/−*^ mice were tail vein injected with Ad-Ripk2 to overexpressed Ripk2 before LPS treatment. Analysis of cytokines and physiological indexes in serum revealed that pre-treating mice with Ad-Ripk2 could prevent sepsis in *Card9*^*−/−*^mice (Fig. 7A, B). The H&E-staining of intestines displayed the reduced lesion area and the recovery of the intestinal barrier in Ad-Ripk2 treated *Card9*^*−/−*^-sepsis mice compared with *Card9*^*−/−*^-sepsis mice (Fig. 7C, D). Similarly, the expression of ZO-1, Occludin, Claudin-2, detected by immunohistochemistry, was increased in the intestinal tissue in *Card9*^*−/−*^-sepsis mice treated with Ad-Ripk2 compared with *Card9*^*−/−*^-sepsis mice (Fig. 7E). We also observed that sepsis in WT mice was reduced after Ad-Ripk2 treatment (Fig. 7A, B). Notably, Ripk2 acts as a downstream molecule of Card9 from the data in Fig. 5C–F. And as shown in Fig. 6A–E, Ripk2 itself was inhibited NLRP3 inflammasome activation in vitro. These results suggested that Ripk2 plays negative regulation in NLRP3 inflammasome activation during sepsis, which is enhanced by Card9.

## Discussion

The systemic effect of sepsis has been well-studied, and the evidence of local changes in the intestinal mucosal cavity influence the course of sepsis has gradually established. Although the pathogenesis of sepsis is complex, a mass of studies have indicated that the destruction of the intestinal barrier may lead to fatal sepsis and multiple organ failure [29]. Card9 is a CARD-containing adaptor protein that plays a role in innate immune signals [30–32]. In this study, we validated Card9 as a negative regulation factor of sepsis. This is quite different from the function of Card9 in fungal signal transduction, where Card9 plays a central role in driving pro-inflammatory signal transduction responses to infection [30, 33]. The host cell’s fight against infection triggers complex, pathogen-specific signaling events that drives the appropriate inflammatory response. Inflammatory signaling pathway do not work in isolation, but instead of form complex networks linking different pathways through key molecules as hubs. Our study and other published research showed that Card9 is one of the pathway nodes. Card9 regulates Toll-like receptor or C-type lectins receptor [23, 34, 35] depended NF-κB signaling, autophagy and production of ROS [36, 37]. Notably, the specific function of Card9 is different in different cell types, thus controlling the cellular specificity in the host’s response to infection [38].

It is of great significance to ascertain the negative regulation of Card9 in sepsis, because excessive inflammation is the main cause of increased mortality in early sepsis [39]. Genome-wide association studies have showed that functional loss of Card9 is related to the development of inflammatory disease and cancer [40–43]. Moreover, Card9-deficient mice displayed higher bacterial burden and increased systemic inflammation after infected with mycobacterium tuberculosis [44]. These observations highlight Card9 acts as a negative regulator for inflammation.

**Fig. 8:**
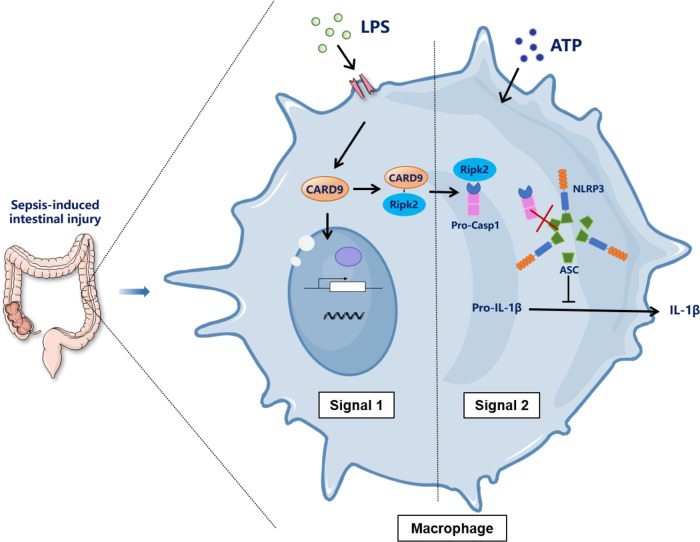
A working model to illustrate how Card9 regulated NLRP3 inflammasome activation. LPS stimulated Card9 to recruit Ripk2 during “signalling 1”, followed by Ripk2 disrupt the assembly of ASC/Caspase-1 by competitively binds with Caspase-1 in “signalling 2”. It acts as a balance regulator to protect the intestinal damage caused by sepsis.

The NLRP3 inflammasome plays an important role in inflammatory and diseases [12], but the mechanism of NLRP3 inflammasome activation need further study. NLRP3 inflammasome signaling includes priming and activation. Previous research indicated that during “Signal 1”, Card9 specifically reduced transcription of NF-κB target genes, pro-IL-1β [26]. Our research demonstrated an effect of Card9 during “Signal 2” of inflammasome activation. As an adaptor protein, Card9 regulates intracellular signaling by interacting with other signaling proteins. Card9 interaction network analysis showed that the interaction between Card9 and Ripk2 was enhanced after LPS stimulation. Previous studies have shown that Ripk2 acts as a key regulator of Caspases-11-dependent non-canonical NLRP3 inflammasome activation [45]. We suggested that Ripk2 suppressed NLRP3 inflammasome activation in macrophages by competitively binds with Caspase-1. Notably, the interaction between Card9 and Ripk2 only enhances the function of Ripk2 on NLRP3 inflammasome activation, and Ripk2 acts as a downstream molecule of Card9. Summarized in Fig. 8, Card9 interacts with and Ripk2, a molecule that disrupt the assembly of ASC/Caspase-1 by competitively binds with Caspase-1 during NLRP3 activation. The molecular occurrence acts as a balance regulator that avoid overactivation of NLRP3 inflammasome and the subsequent overproduction of IL-1β and IL-18. In addition, as a downstream molecule of Card9, high expression of Ripk2 can alleviate septic damage caused by Card9 loss.

Together, our data reveals a novel mechanism by which Card9, through recruiting Ripk2, provides a crucial negative regulation to prevent uncontrolled activation of NLRP3 inflammasome.

## Materials and methods

### Mouse model

Wild-type (WT) mice were purchased from Institute of Model Animals, Nanjing University. *Card9*^*−/−*^ mice were provided by Dr. Xin Lin (Tsinghua University, Beijing, China). Mice were raised in sterile facilities at the Medical College of Nanjing University. All animal experiments were performed according to NIH guidelines for “The Care and Use of Laboratory Animals” and approved by the Institutional Committee of Animal Care and Use of Medical College of Nanjing University. Animals were randomly separated into four groups (*n* = 10 in each group). WT Ctrl and Card9^-/-^ Ctrl groups mice were intraperitoneally treated with saline solution (0.9% NaCl). WT-sepsis and *Card9*^*−/−*^-sepsis groups was intraperitoneally treated with ultrapure Escherichia coli O111:B4 strain LPS (Sigma-Aldrich, #L2630) 5 mg/kg for 24 h.

### BMDMs generation and stimulation

Primary bone marrow cells were isolated from mouse bone marrow, culturing cells in the DMEM medium which added M-CSF (20 ng/ml). After 7 days, BMDMs were priming with 100 ng/ml LPS for 3 h, and then stimulated with ATP (5 mM, 1 h), MSU (500 μg/ml, 2 h) or Nigericin (10 µM, 2 h), respectively. To activate the AIM2 inflammasome, BMDMs were primed with LPS as above, followed by transfection of poly(dA-dT) (2 μg/ml) using Lipofectamine 2000 (Life Technologies) according to manufacturer’s protocol. To activate the NLRC4 inflammasome, cells were primed with LPS followed by transfected with 1 µg/mL Flagellin (Sigma-Aldrich, # SRP8029) for 6 h.

### MACS cell separation

Anti-F4/80 MicroBeads UltraPure (Miltenyi Biotec, #130-110-443) were used to isolating of macrophages from the intestinal tissue according to manufacturer’s instructions.

### Serology, cytokines and Caspase-1 activation measurement

Blood samples were collected 24 h after LPS or saline solution injection. The blood samples were centrifuged at 4000 rpm for 15 min to separating serum. All serum samples were analysed within 24 h. AST and ALT levels were measured with BioAssay Systems Kit (BioAssay Systems) according to company’s protocol. Concentration of IL-1β, IL-10, TNF-α, IL-6 were evaluated with the murine ELISA kit (MULTI SCIENCES, China). Percentage of Caspase-1 activation was detected by Caspase-1 Fluorometric Assay Kit (abcam, # ab39412) according to manufacturer’s instructions.

### RNA isolation and qPCR

RNA extraction was using FastpPure Cell/Tissue Total RNA Isolation Kit (Vazyme, #RC112-01). RNA concentration and purity were determined by Nanodrop2000. HiScript RT SuperMix (Vazyme, #R323-01) was used to synthesis cDNA. qPCR was using SYBR Green Master Mix (Appliedbiosystems, #A25742), the machine was Applied Biosystems with Viia 7 software. The primer sequence is as follows: IL-1β (F:5’-GAAATGCCACCCTTTTTACAGTG-3’; R: 5’-TGGATGTCTCTTCATCAGGACAG-3’); IL-18 (F:5’-GTGAACCCCAGACCAGACTG-3’; R:5’-CTGGAACACGTTTCTGAAAGA -3’).

### Isolation of spleen cells and flow cytometry

Spleen and colon were isolated, grinded, lytic erythrocyte and washed. The cell suspension was filtered through a sieve, and then centrifuged at a speed of 1300 rpm. Cells were stained with following antibodies purchase from Biolegend for 15 min: CD11b (FITC, #101205), F4-80 (PE, #123109), CD3 (FITC, #100203), CD8 (PE, #100707), CD4 (PE, #100407), LY6G (APC, # 127613).

### Protein isolation and western blotting

Protein concentration was determined by the BCA protein assay [43]. Membranes were exposed to the following antibodies purchased from Cell Signaling Technology: Occludin (#91131), Claudin-2 (#48120), Cleaved-IL-1β (#63124), IL-18 (#57058), Caspase-1 (#2225), NLRP3 (#15101), ASC/TMS1 (#67824), AIM2(#63660), β-actin (#4970), Malt1(#2494), Bcl10(#4237), Nod1(#3545), Card9 (#12283), Ripk2 (#4142). Anti-ZO-1 antibody purchased from abcam (ab221547), Anti-NLRC4 antibody purchased from ECM Bioscience(#NP5381). Western blotting signals were quantified by a FluorChem densitometer (Alpha Innotech, San Leandro, CA).

### Immunofluorescent staining and confocal microscopy

The intestinal tissue and cells were fixed in 2% paraformaldehyde (PFA) for 15 min and immunofluorescence assay was performed as previously described [46].

### Immunoprecipitation and co-immunoprecipitation

Immunoprecipitation and co-immunoprecipitation experiments were conducted utilizing Pierce Classic Magnetic IP/Co-IP Kit (Thermo Scientific, #88804). Protein lysates followed by immunoblotting with following antibody.

### Small Interfering RNA (siRNA) and plasmid

For siRNA transfection, one day before transfection, 5 **×** 10^4^ BMDMs were inoculated on 24-well plates with a density of about 50%. Then, BMDMs were transfected with siRNA-Ripk2 or negative control siRNA (siRNA-Ctrl) (RiboBio, Guangzhou, China) using X-tremeGENE siRNA Transfection Reagent (Roche, #04476115001) according to manufacturer’s instructions. siRNA and X-tremeGENE siRNA Transfection Reagent were diluted with opti-MEM I medium. The ratio of X-tremeGENE siRNA Transfection Reagent (μl) to siRNA (μg) is 5 to 1. The gene silencing efficiency was evaluated by Q-PCR 72 h after transfection.

### Generation of recombinant adenoviruses and transfection

The mouse Ripk2, Card9, Caspase-1 and ASC gene sequence was available from GenBank. The mouse Ripk2 sequence was subcloned into an adenovirus shuttle plasmid vector: pLV-CMV-MCS-HA-IRES-neo. The mouse Card9 or ASC sequence was subcloned into an adenovirus shuttle plasmid vector: pLV-hef1a-mNeongreen-P2A-neo-WPRE-CMV-MCS-3flag. The mouse Caspase-1 sequence was subcloned into an adenovirus shuttle plasmid vector: CMV-MCS-EF1α-ZsGreen1-PGK-Puro (PHY-028). HA-Ripk2, Flag-Card9 and V5-Caspase-1 were manufactrured by Hesheng Technology (China). Bone marrow-derived macrophages were transfection in 5 ml of DMEM with 2% FCS containing recombinant adenovirus. The cells were incubated at 37 °C/5% CO_2_ for 90 min with rocking every 15 min after which the medium was replaced with full DMEM.

### Adenovirus-mediated gene transfer of Ripk2 in vivo

To evaluate the efficacy of the Ripk2 gene transfer in mice, male 8-week-old wild-type mice and *Card9*^*−/−*^ mice (*n* = 10) were tail vein injected with Ad-Ripk2 (5 × 10^8^ plaque-forming units) for 24 h before LPS treatment.

### Statistical analysis

Statistical differences between groups were determined by the Student’s *t*-test and two-way ANOVA test. Differences were considered significant at *P* < 0.05. No data points or mice have been ruled out. All statistical analyses were performed using Prism 8.0 (GraphPad Software, CA).

## Supplementary information

Supplementary Figures

Original Data File

Reproducibility checklist

## Data Availability

All data needed to evaluate the conclusions in the paper are present in the paper. Additional data related to this paper may be requested from the corresponding author.
