# Supplementary material for: Card9 protects sepsis by regulating Ripk2-mediated activation of NLRP3 inflammasome in macrophages
Source: Cell Death Dis. 2022 May 26;13(5):502. doi: 10.1038/s41419-022-04938-y (PMC9135688; doi:10.1038/s41419-022-04938-y)
Supplement: Supplementary file 1 — Supplementary Figures [file 41419_2022_4938_MOESM1_ESM.docx]

**Card9 protects sepsis by regulating Ripk2-mediated activation of NLRP3 inflammasome in macrophages**

Zhen Xu^1,2^, Daoqian Li^1,2^, Wei Qu^1,2^, Yuxin Yin^1,2^, Shuping Qiao^1,2^, Yanan Zhu^3^, Sunan Shen^1,2^, Yayi Hou^1,2*^, Jie Yang^4*^, Tingting Wang^1,2*^

**Supplementary Figures.**

**
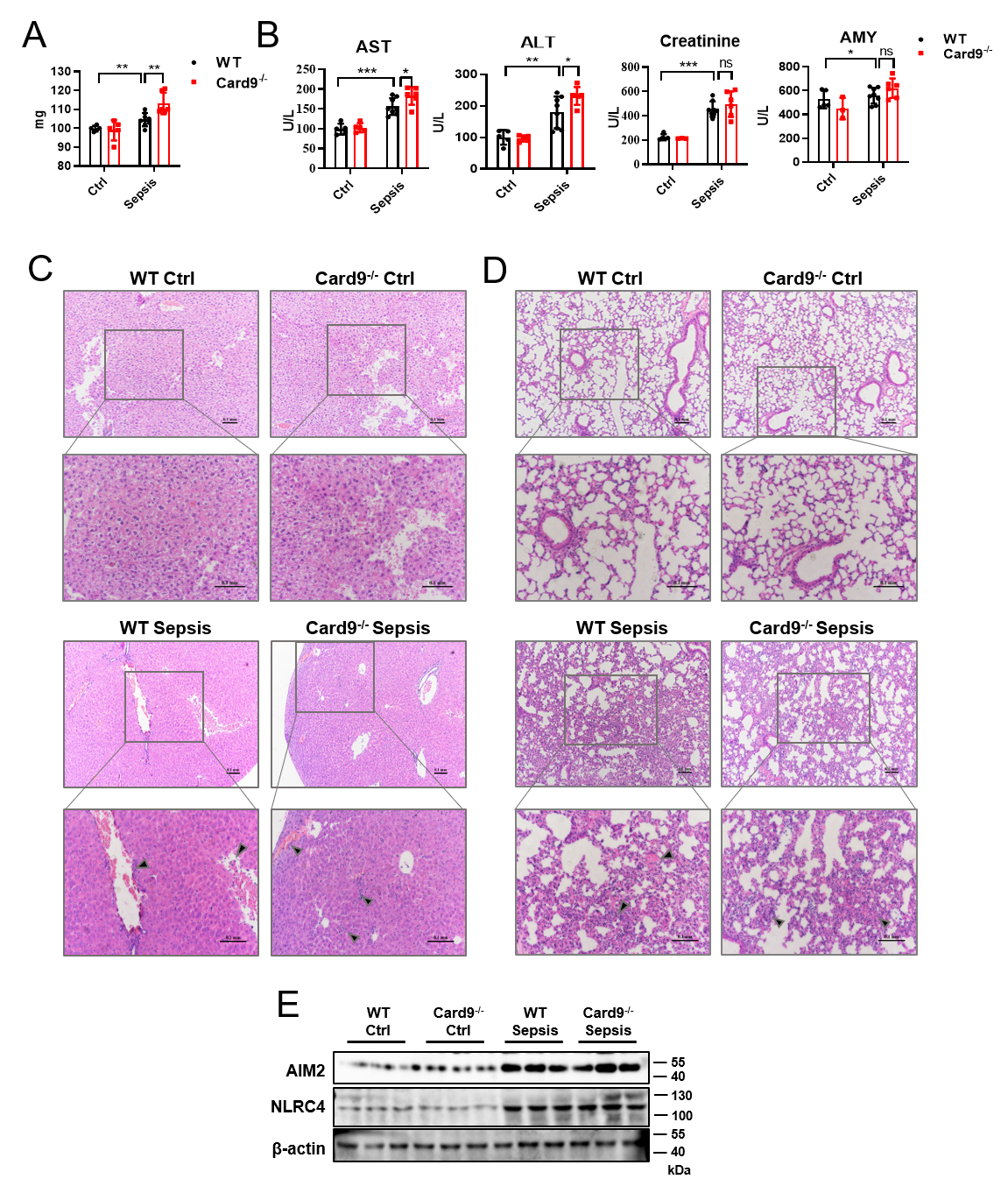
**

**Supplementary Figure 1. The liver and lungs injury in *Card9^-/-^* mice.**

(A) Spleen weight of WT mice and *Card9^-/-^* mice. (B) Production of creatinine, AST, ALT, Creatinine and AMY was detected in serum. (C-D) Histological analysis of liver and lung. (E) Western blot analysis of AIM2 and NLRC4 in intestinal tissue from WT mice and *Card9^-/-^* mice. Data are shown as mean ± SEM. Each group was representative of at least three biological replicates. *p < 0.05, **p < 0.01, ***p < 0.001.


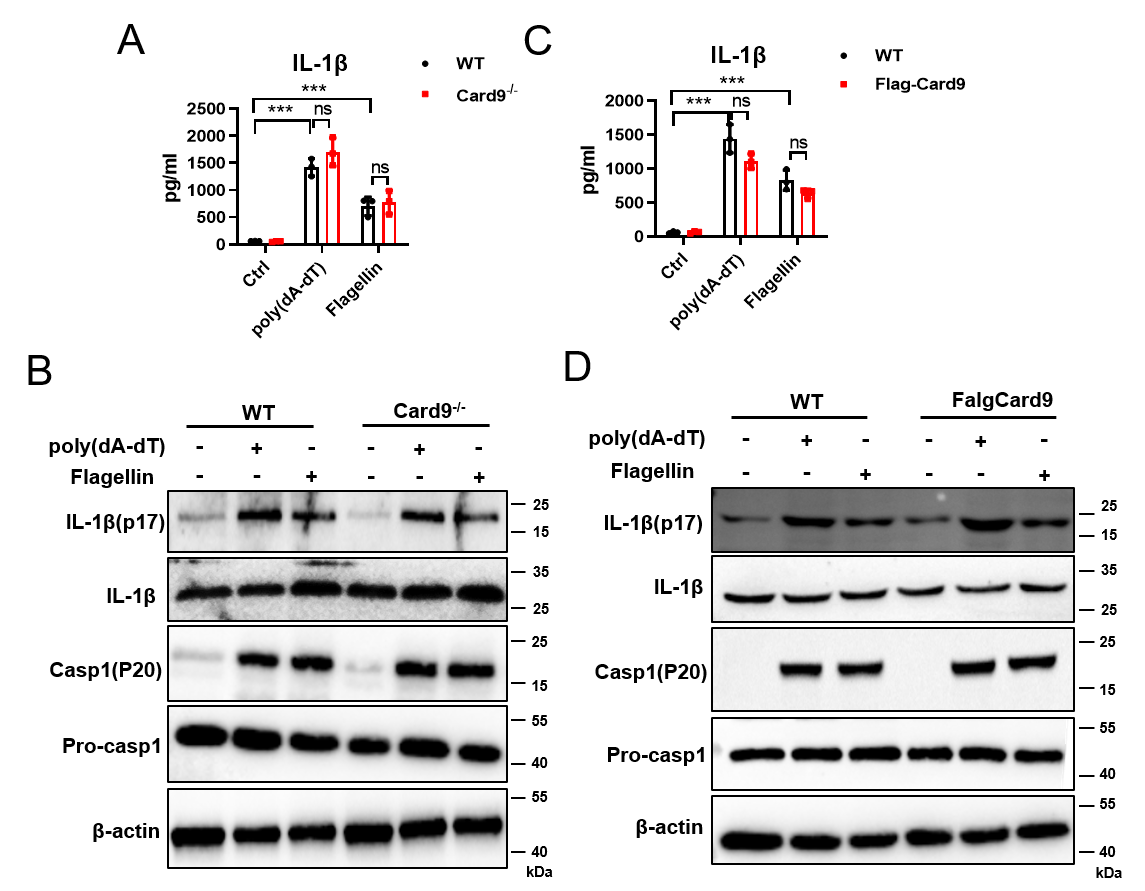


**Supplementary Figure2.** **Card9 is dispensable for AIM2 and NLRC4 inflammasome activation.**

(A-B) BMDMs were obtained from WT mice and *Card9^-/-^* mice. Cells were unstimulated (medium) or pretreatment with LPS (100ng/ml) for 3 h, followed by transfection of poly(dA-dT) (2μg/ml) or treated with 1 µg/mL Flagellin for 6 h. (A) ELISA analysis of IL-1β secretion in the culture supernatant. (B) Western blot analysis of cell lysates. (C-D) BMDMs were transfected with Card9 over-expression plasmid. Cells were unstimulated (medium) or pretreatment with LPS (100ng/ml) for 3 h, followed by transfection of poly(dA-dT) (2μg/ml) or treated with 1 µg/mL Flagellin for 6 h. (C) ELISA analysis of IL-1β secretion in the culture supernatant. (D) Western blot analysis of cell lysates. Each group was representative of at least three biological replicates. ***p < 0.001.


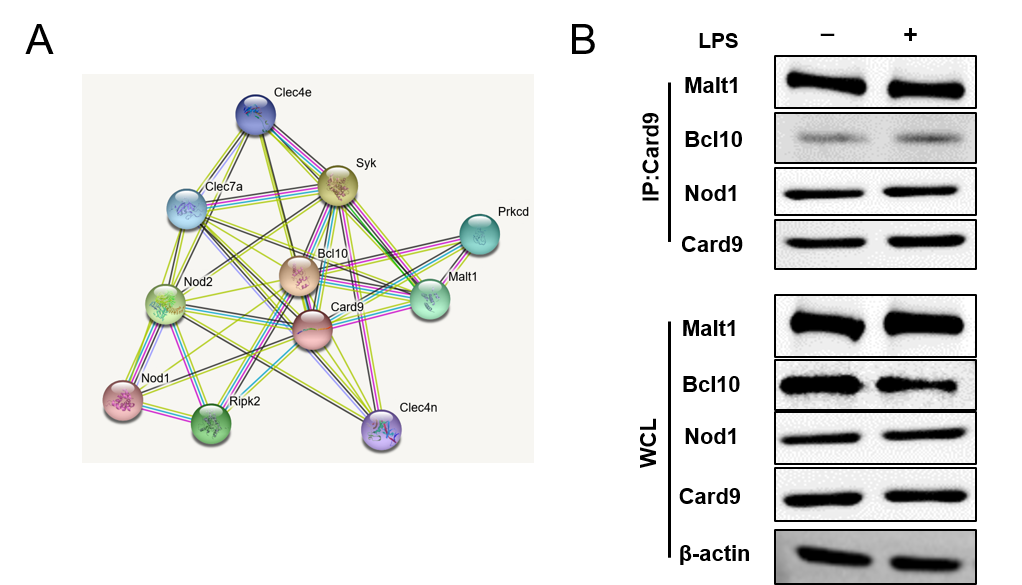


**Supplementary Figure 3. The protein interaction network of Card9.**

(A) STRINGv10 was used to perform functional association network analysis of the main Card9 interactome. High-trust interaction partners in mice (score > 0.7). (B) Western blot analysis of Card9 interactome in BMDMs treated with LPS using co-immunoprecipitation (Co-IP). Data are shown as mean ± SEM. Each group was representative of at least three biological replicates.
