# Supplementary material for: Card9 protects sepsis by regulating Ripk2-mediated activation of NLRP3 inflammasome in macrophages
Source: Cell Death Dis. 2022 May 26;13(5):502. doi: 10.1038/s41419-022-04938-y (PMC9135688; doi:10.1038/s41419-022-04938-y)
Supplement: Supplementary file 2 — Original Data File [file 41419_2022_4938_MOESM2_ESM.pdf]

Figure1F

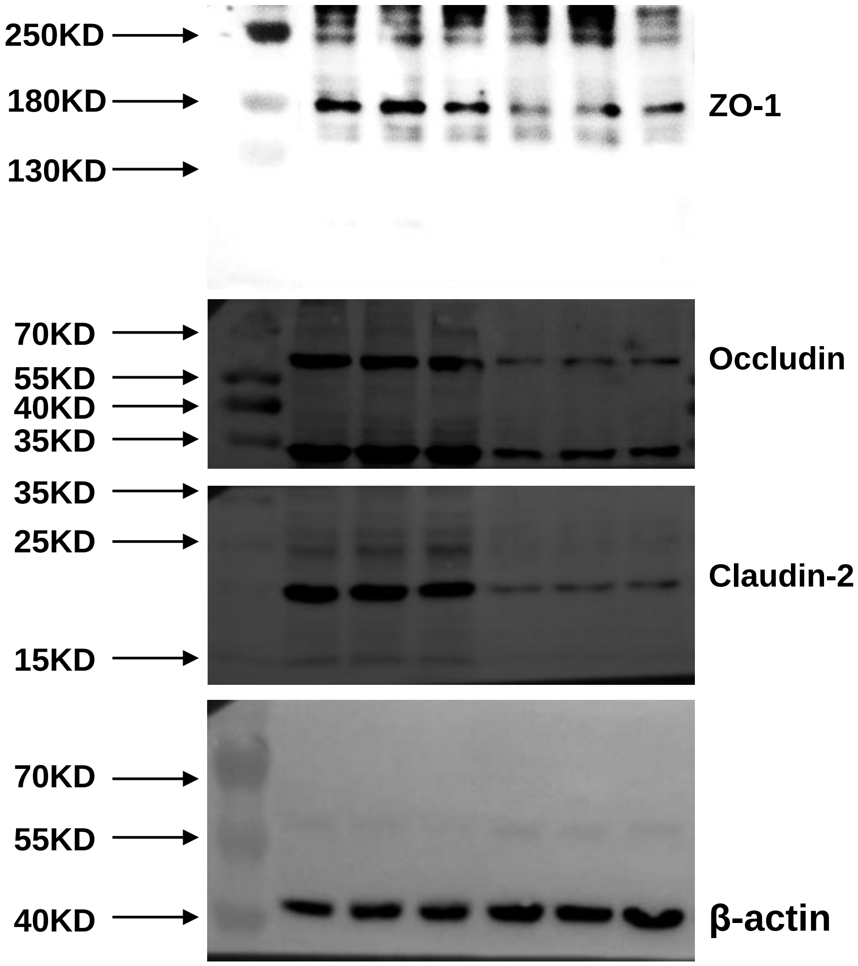
250KD
180KD
130KD
ZO-1
70KD
55KD
40KD
35KD
Occludin
35KD
25KD
15KD
Claudin-2
70KD
55KD
40KD
β-actin

Figure2D

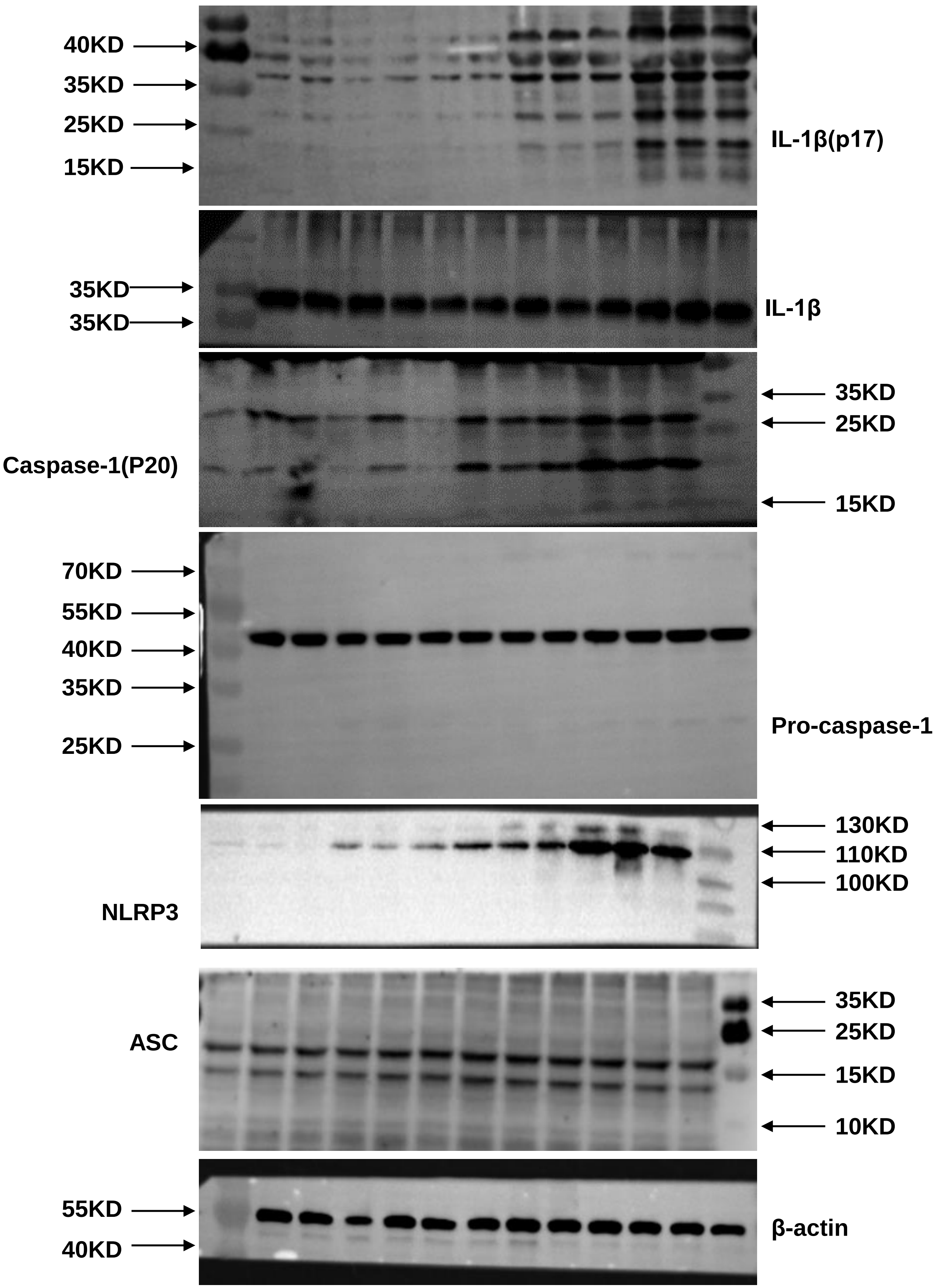
40KD
35KD
25KD
15KD
IL-1β(p17)
35KD
35KD
IL-1β
35KD
25KD
Caspase-1(P20)
15KD
70KD
55KD
40KD
35KD
25KD
Pro-caspase-1
130KD
110KD
100KD
NLRP3
35KD
25KD
ASC
15KD
10KD
55KD
40KD
β-actin

Figure3B

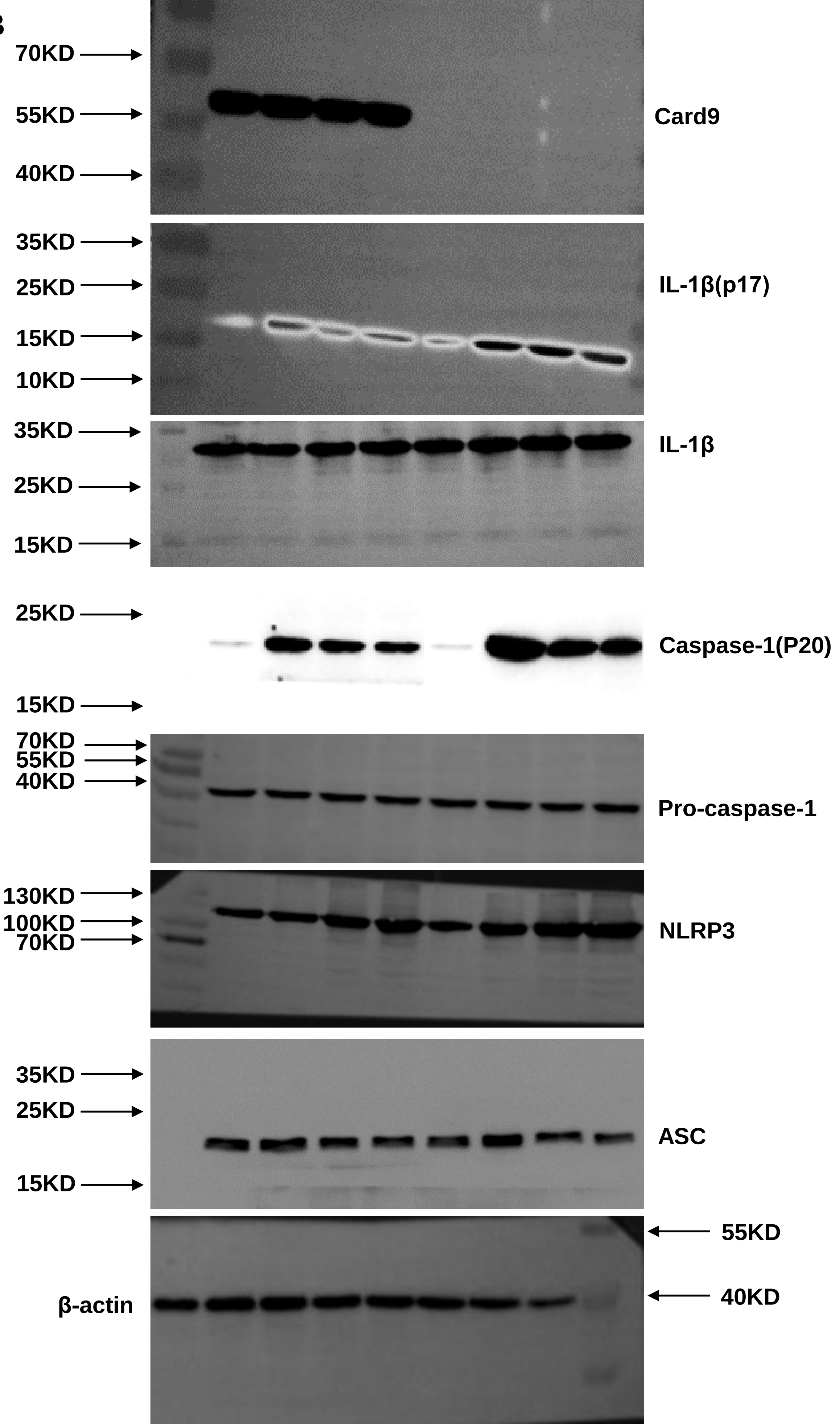
70KD
55KD
40KD
Card9
35KD
25KD
15KD
10KD
IL-1β(p17)
35KD
25KD
15KD
IL-1β
25KD
15KD
Caspase-1(P20)
70KD
55KD
40KD
Pro-caspase-1
130KD
100KD
70KD
NLRP3
35KD
25KD
15KD
ASC
β-actin
55KD
40KD

Figure3C

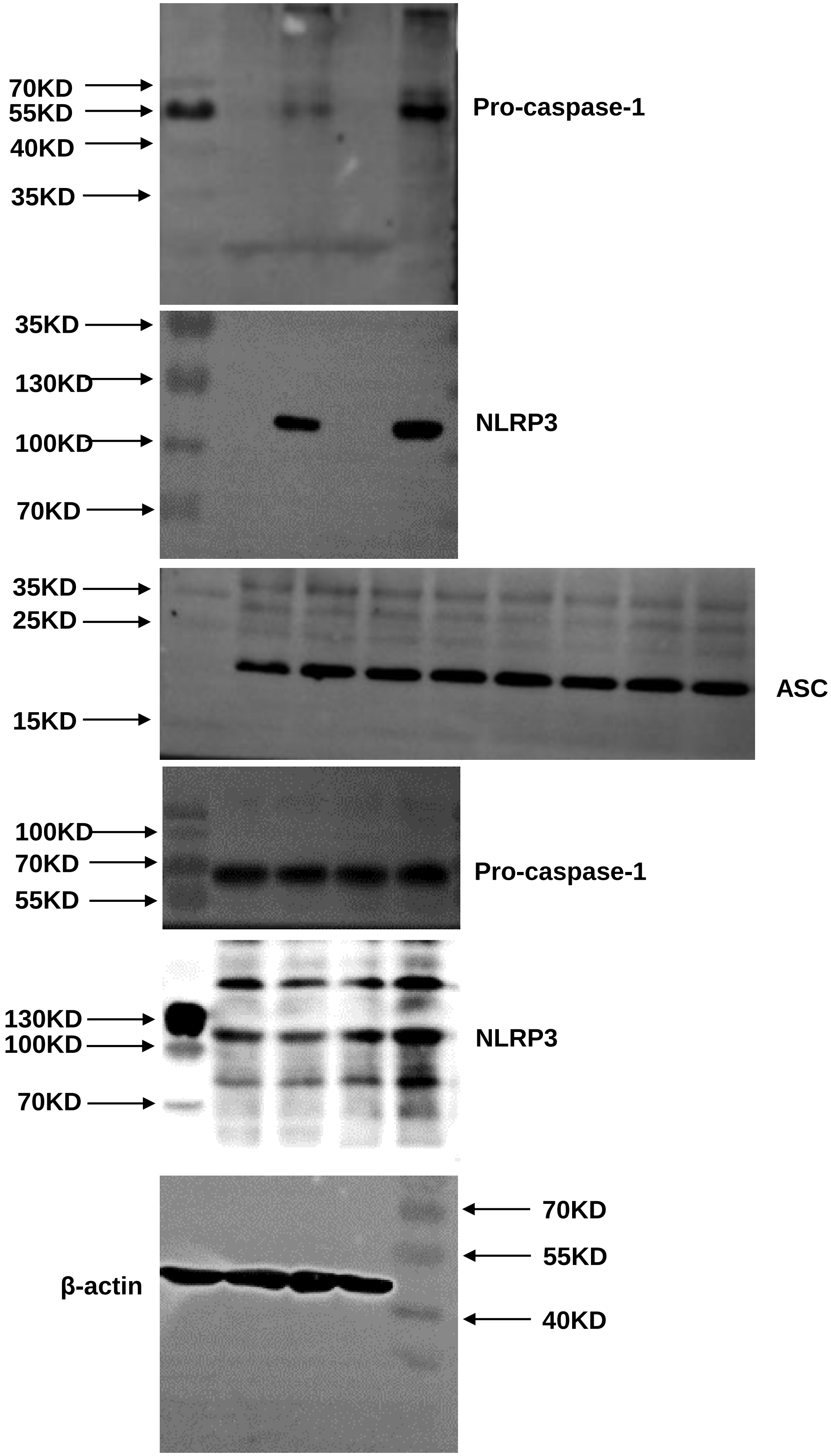
70KD
55KD
40KD
35KD
Pro-caspase-1
35KD
130KD
100KD
70KD
NLRP3
35KD
25KD
15KD
ASC
100KD
70KD
55KD
Pro-caspase-1
130KD
100KD
70KD
NLRP3
β-actin
70KD
55KD
40KD

Figure4B

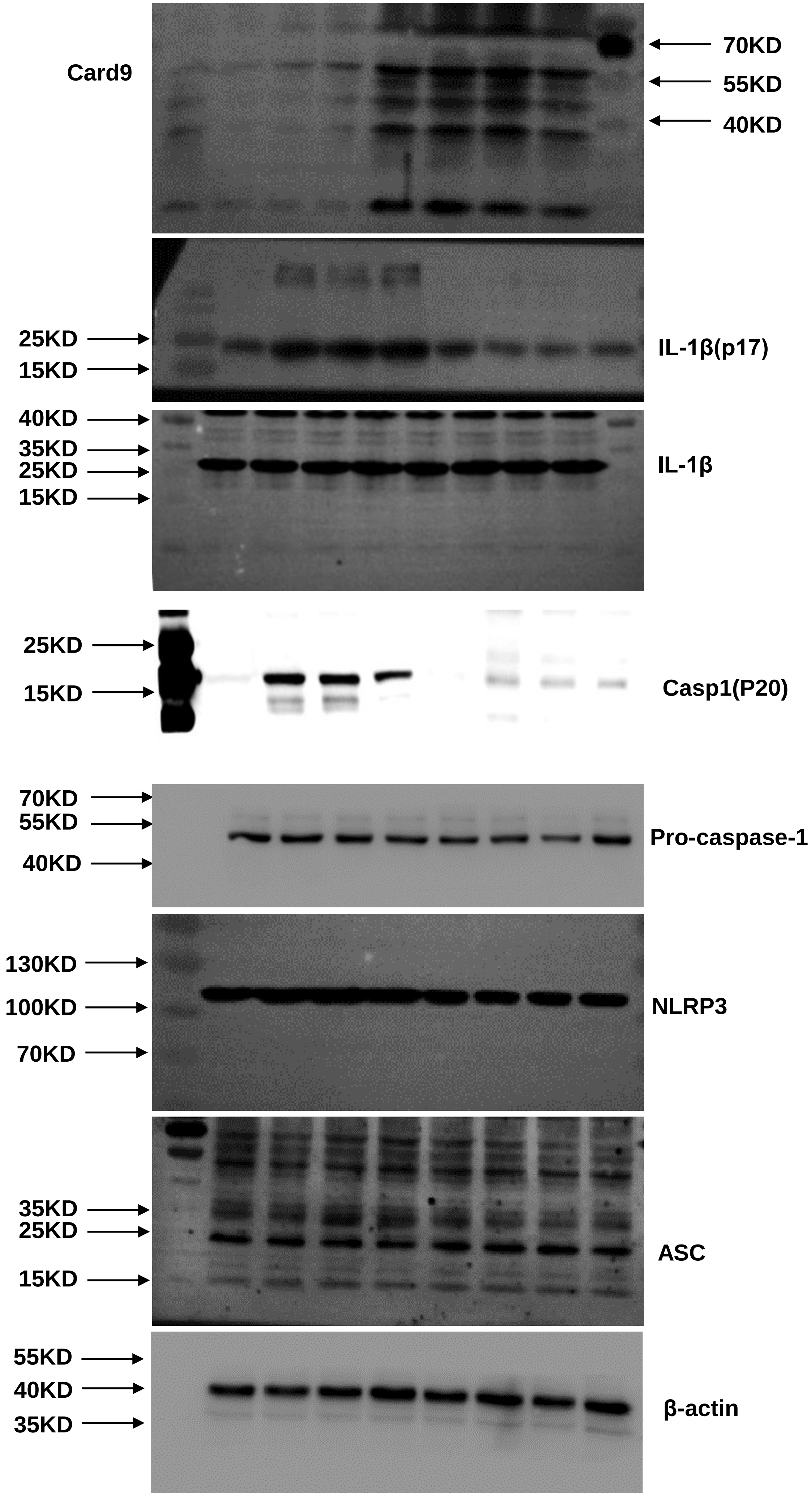
Card9
70KD
55KD
40KD
25KD
15KD
IL-1β(p17)
40KD
35KD
25KD
15KD
IL-1β
25KD
15KD
Casp1(P20)
70KD
55KD
40KD
Pro-caspase-1
130KD
100KD
70KD
NLRP3
35KD
25KD
15KD
ASC
55KD
40KD
35KD
β-actin

Figure4C

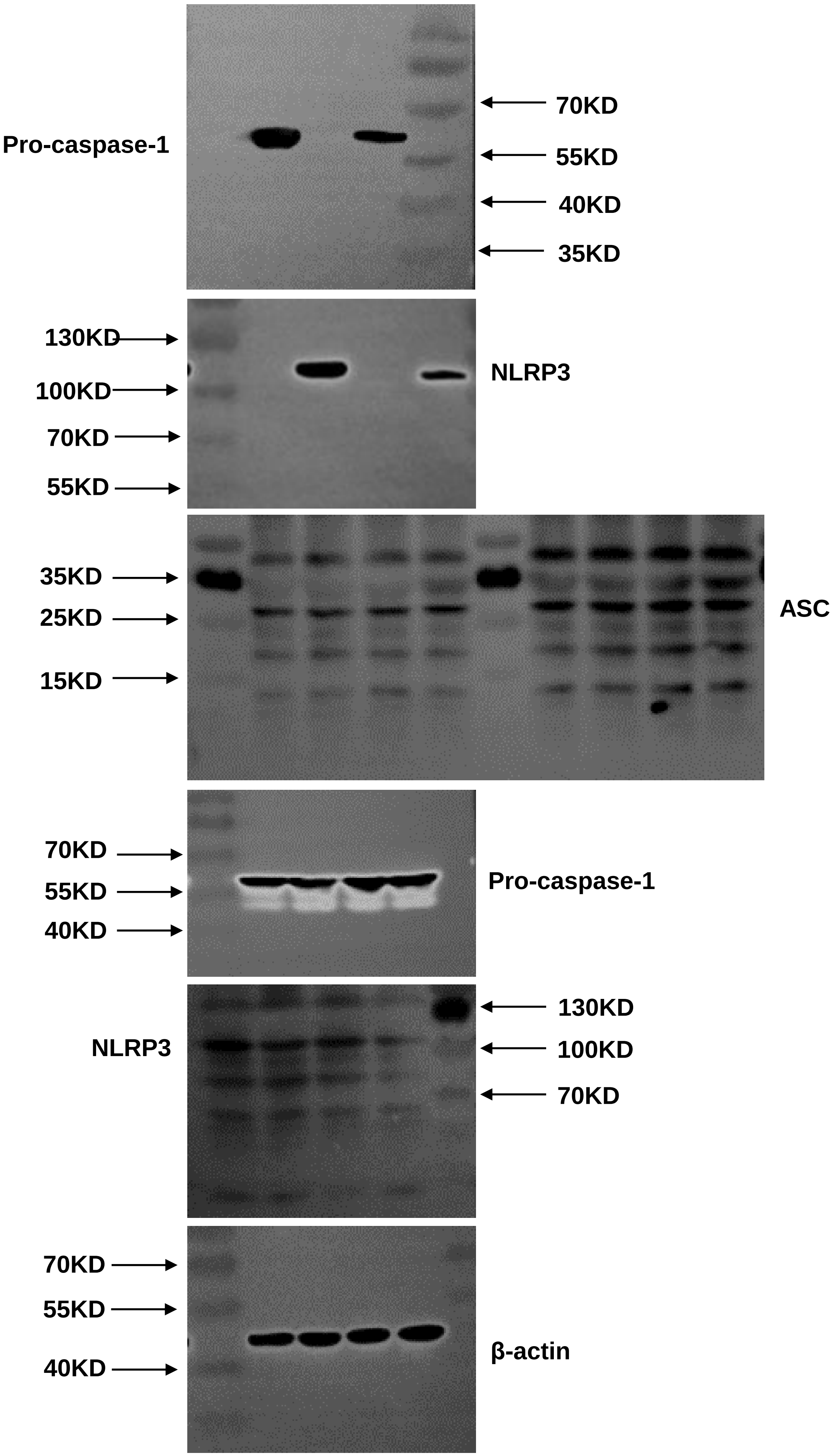

Pro-caspase-1
70KD
55KD
40KD
35KD
130KD
100KD
70KD
55KD
NLRP3
35KD
25KD
15KD
ASC
70KD
55KD
40KD
Pro-caspase-1
NLRP3
130KD
100KD
70KD
70KD
55KD
40KD
β-actin

Figure5A

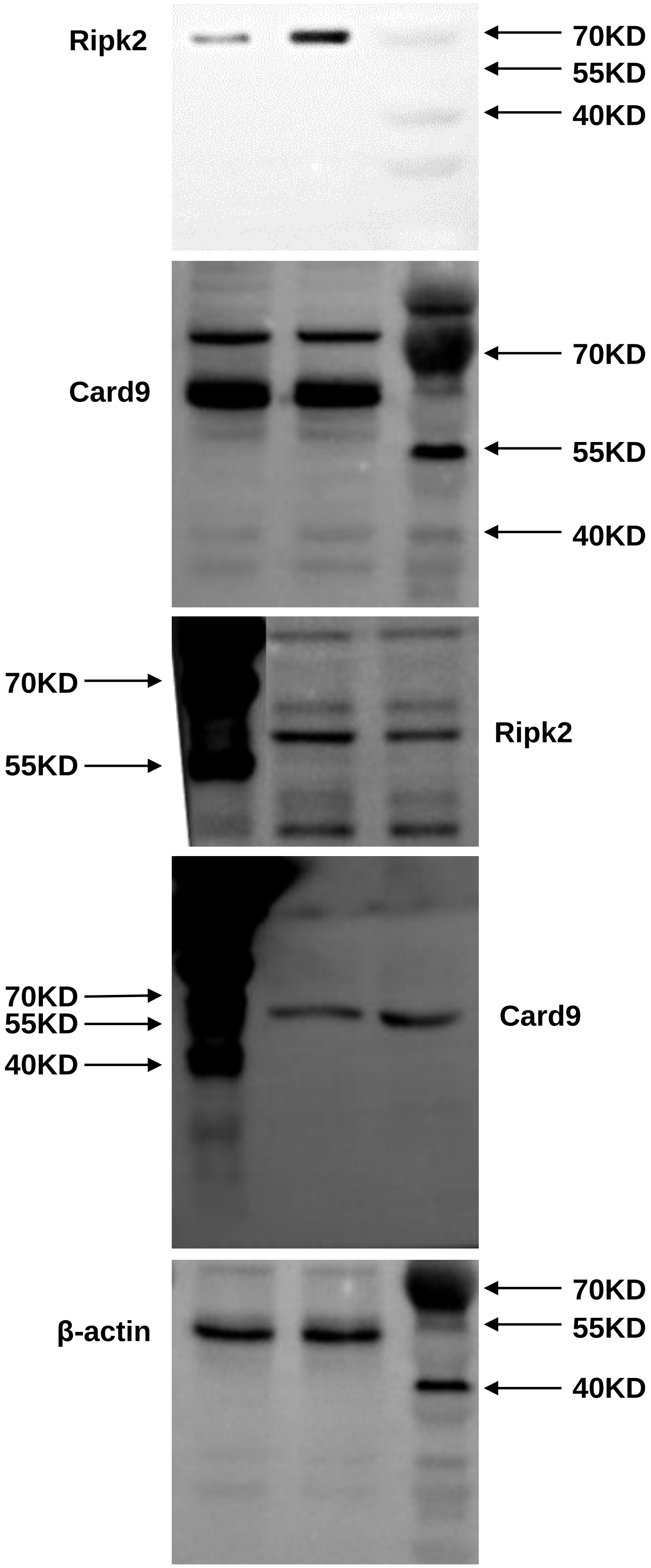
Ripk2
70KD
55KD
40KD
Card9
70KD
55KD
40KD
70KD
55KD
Ripk2
70KD
55KD
40KD
Card9
β-actin
70KD
55KD
40KD

Figure5B

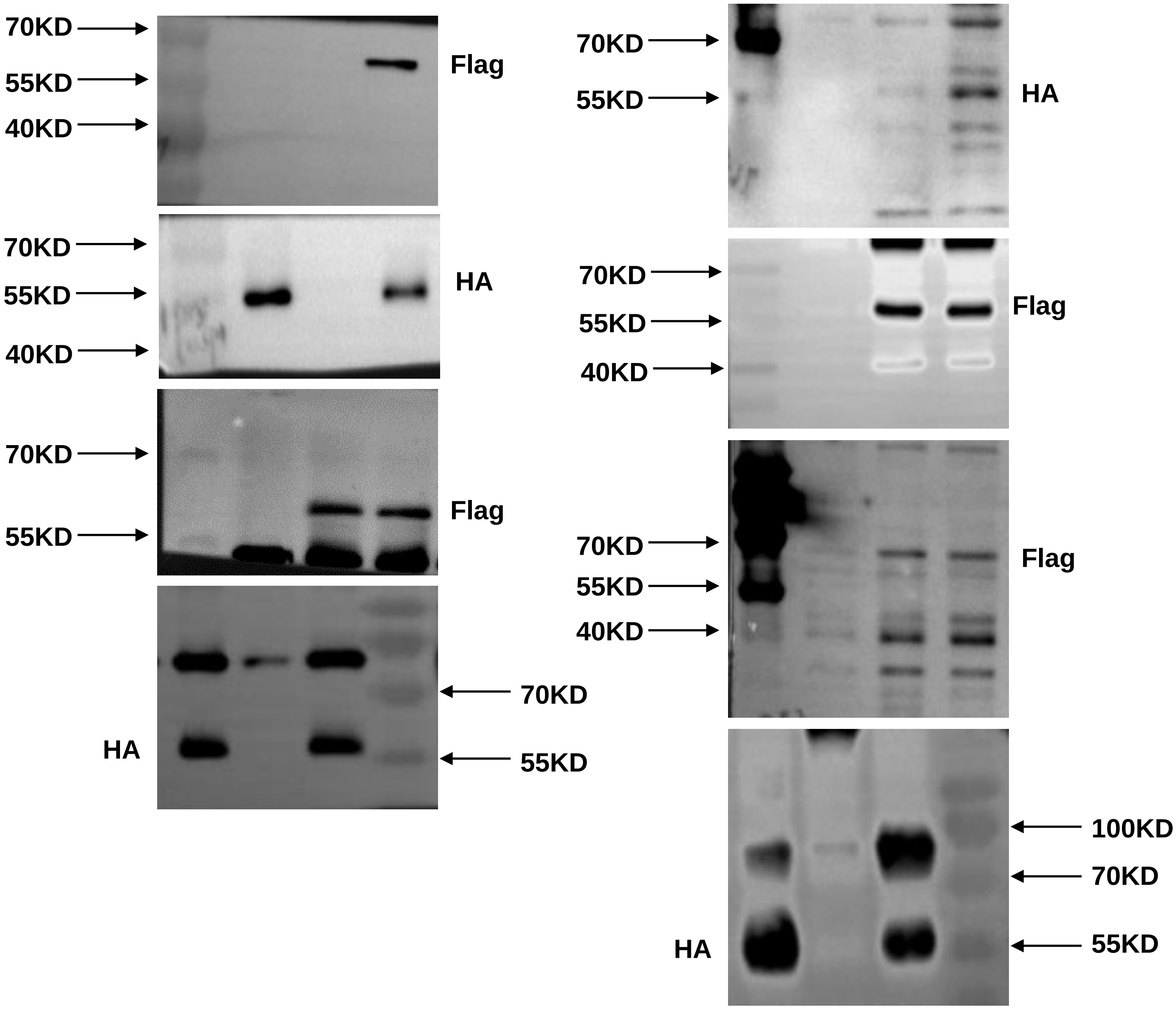
70KD
55KD
40KD
Flag
70KD
55KD
40KD
HA
70KD
55KD
Flag
HA
70KD
55KD
70KD
55KD
HA
70KD
55KD
40KD
Flag
70KD
55KD
40KD
Flag
HA
100KD
70KD
55KD

Figure5D

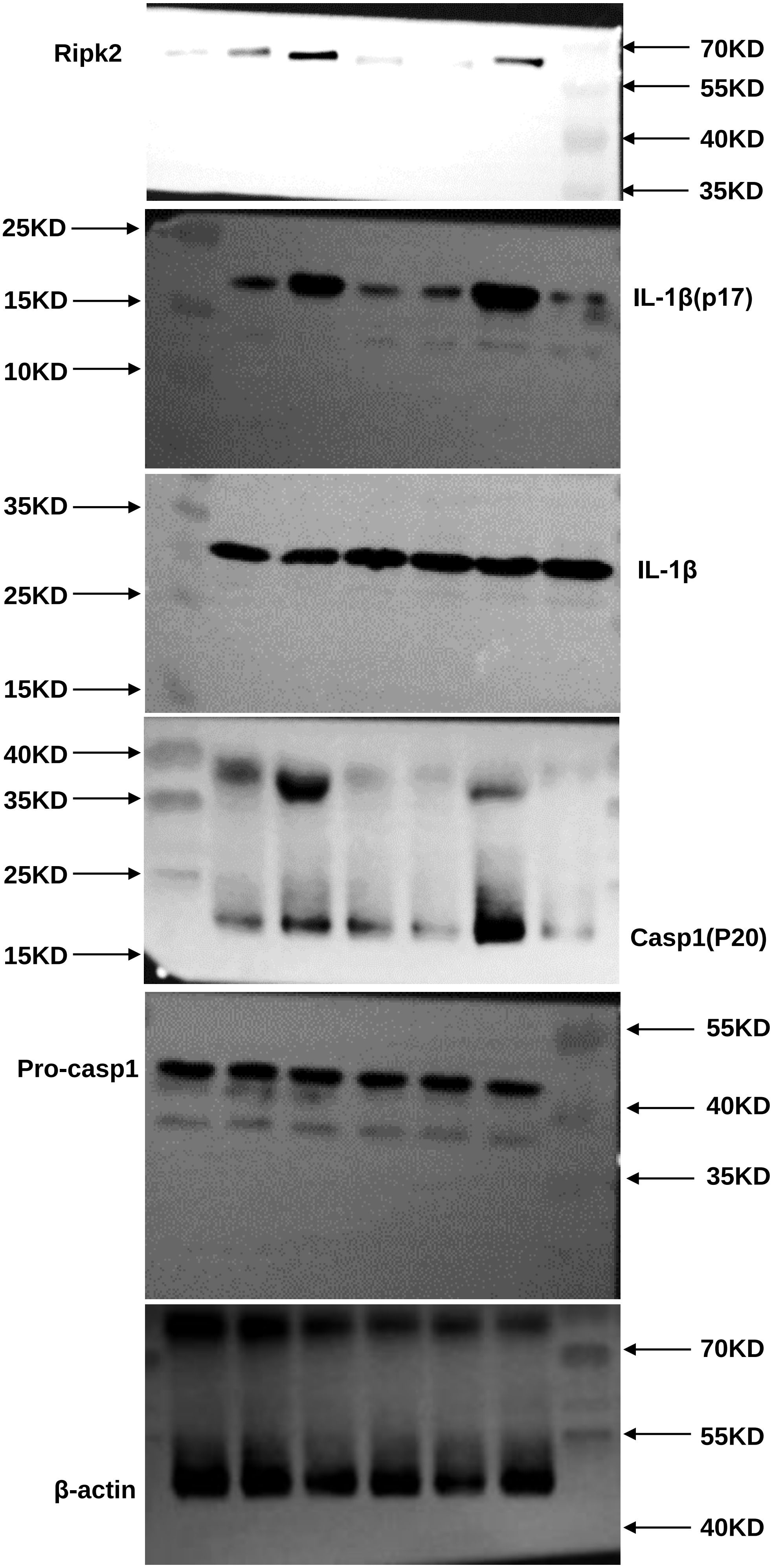
Ripk2
70KD
55KD
40KD
35KD
25KD
15KD
10KD
IL-1β(p17)
35KD
25KD
15KD
IL-1β
40KD
35KD
25KD
15KD
Casp1(P20)
Pro-casp1
55KD
40KD
35KD
70KD
55KD
β-actin
40KD

Figure5F

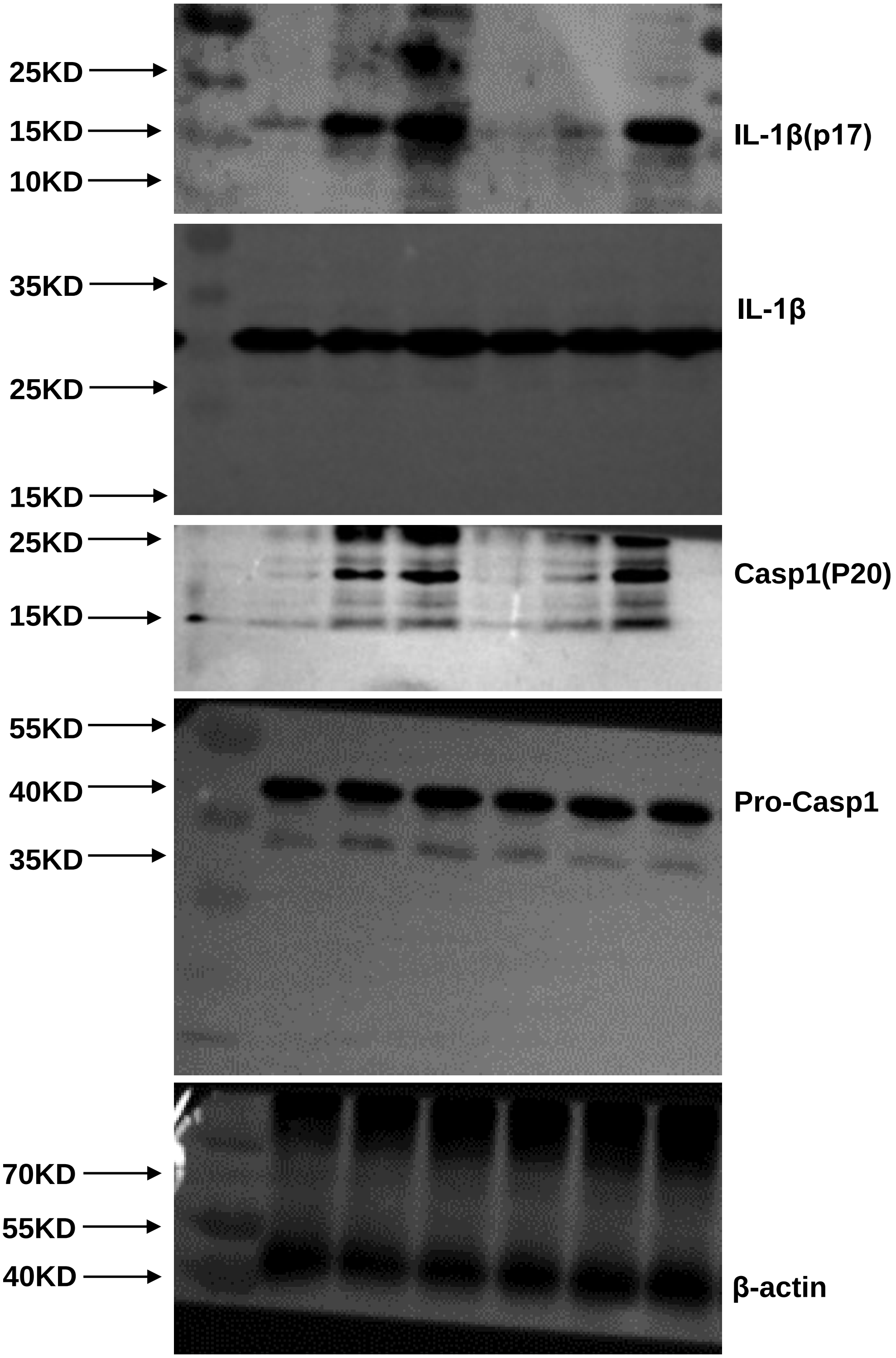

25KD
15KD
10KD
IL-1β(p17)
35KD
25KD
15KD
IL-1β
25KD
15KD
Casp1(P20)
55KD
40KD
35KD
Pro-Casp1
70KD
55KD
40KD
β-actin

Figure5G

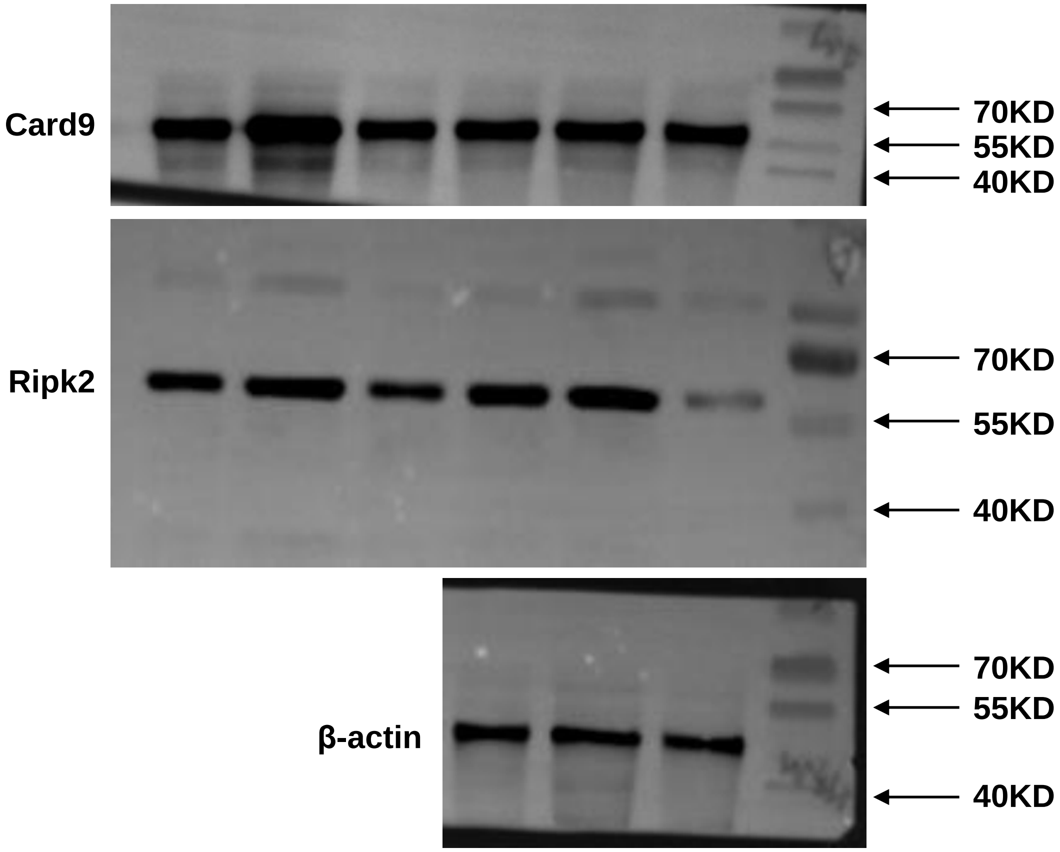

Card9
70KD
55KD
40KD
Ripk2
70KD
55KD
40KD
β-actin
70KD
55KD
40KD

Figure6A

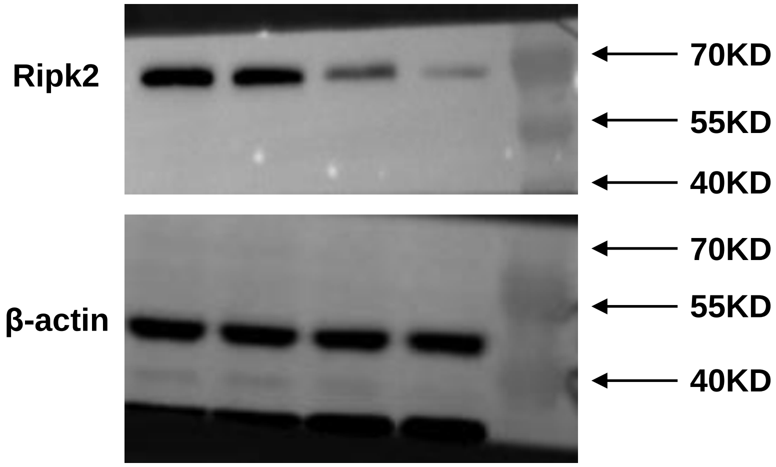

Figure6C

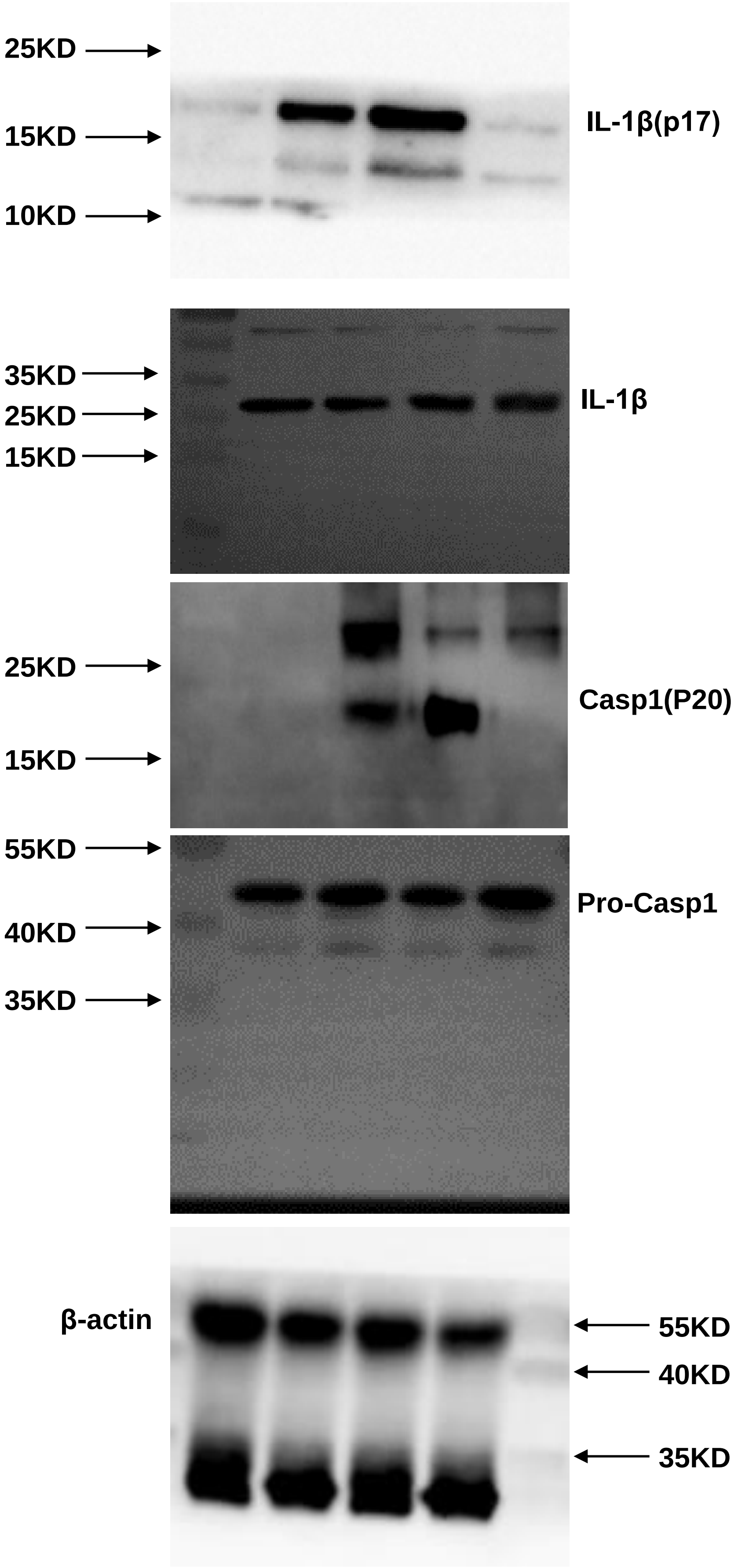
25KD
15KD
10KD
IL-1β(p17)
35KD
25KD
15KD
IL-1β
25KD
15KD
Casp1(P20)
55KD
40KD
35KD
Pro-Casp1
β-actin
55KD
40KD
35KD

Figure6E

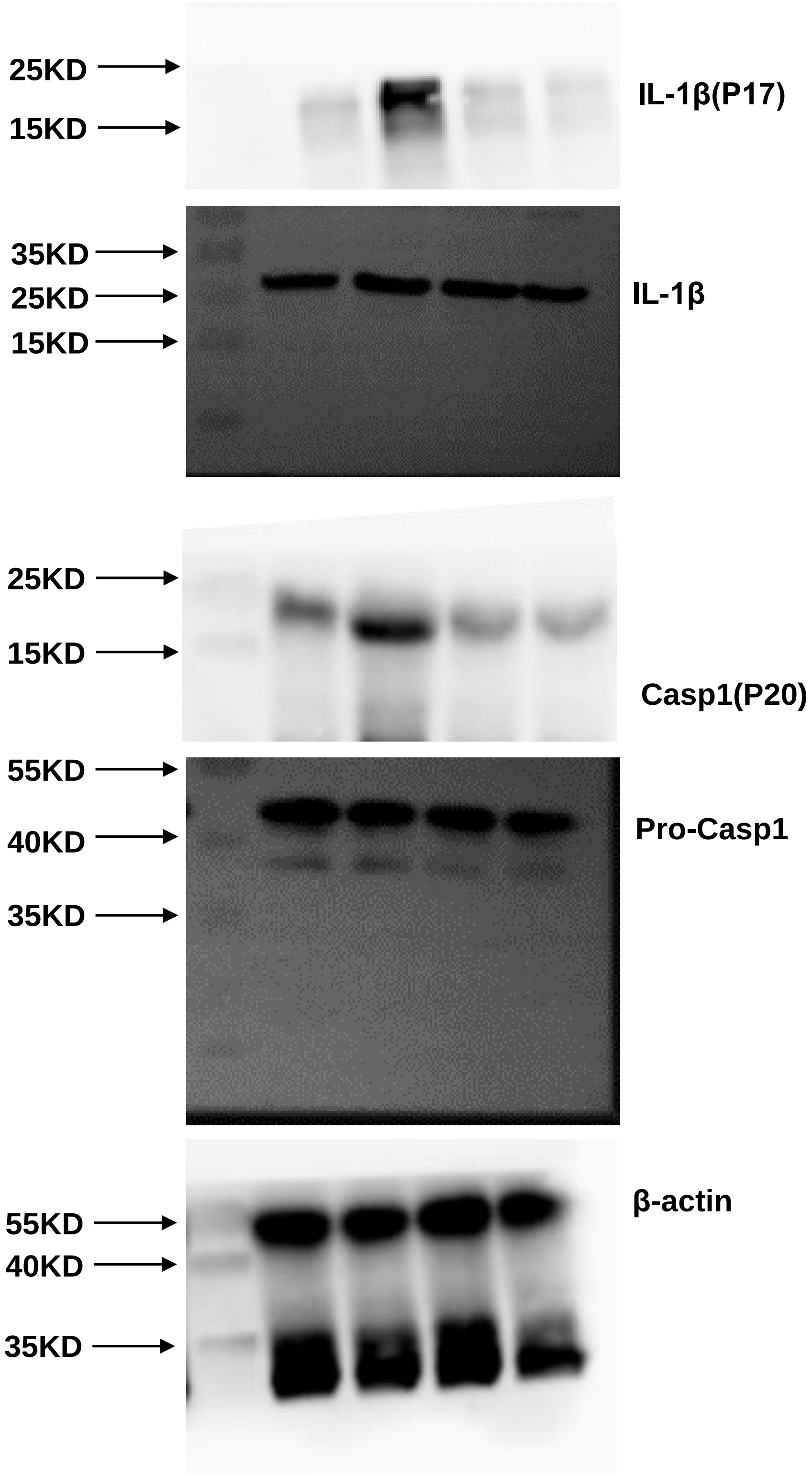
25KD
15KD
IL-1β(P17)
35KD
25KD
15KD
IL-1β
25KD
15KD
Casp1(P20)
55KD
40KD
35KD
Pro-Casp1
55KD
40KD
35KD
β-actin

Figure6F

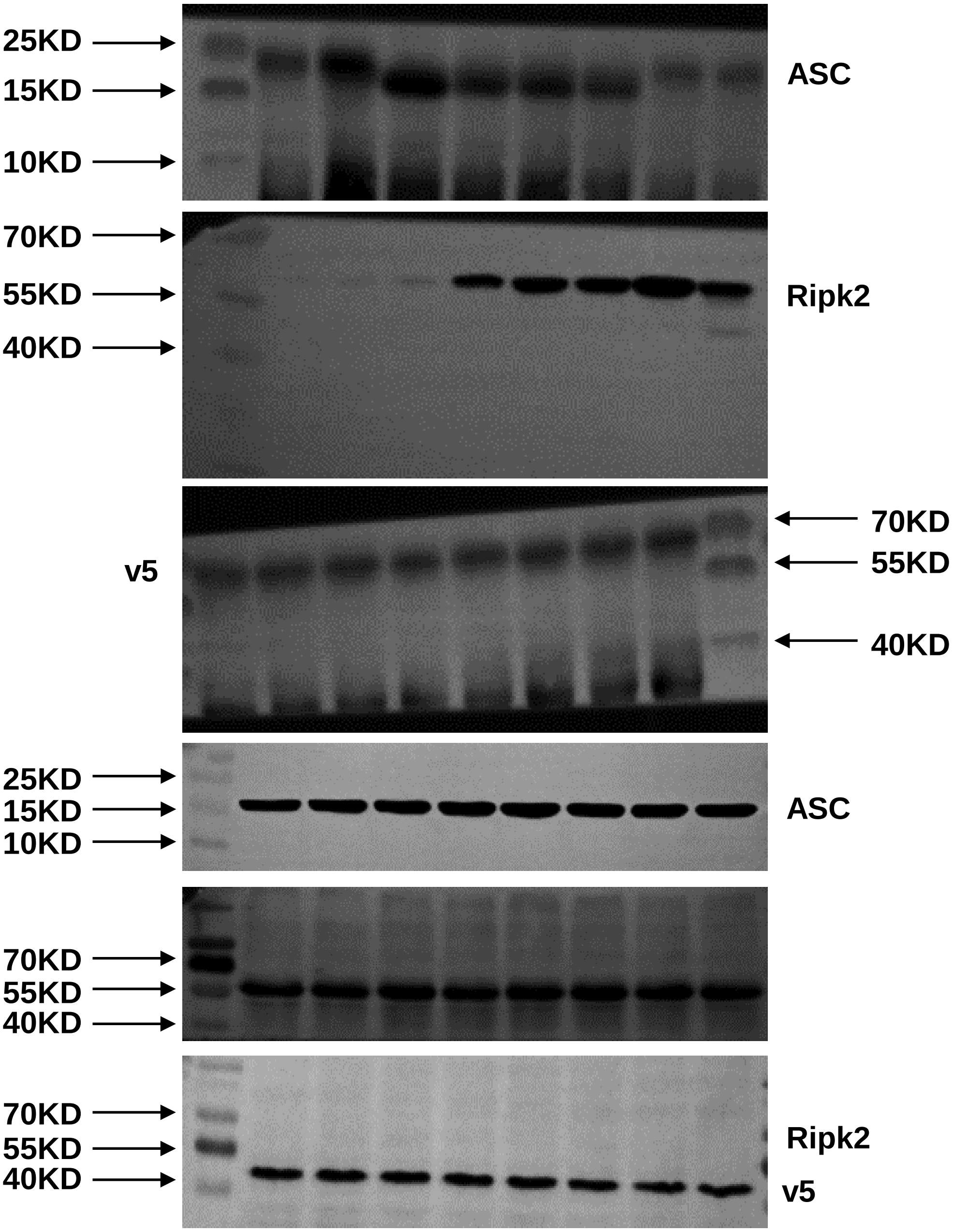
25KD
15KD
10KD
ASC
70KD
55KD
40KD
Ripk2
v5
70KD
55KD
40KD
25KD
15KD
10KD
ASC
70KD
55KD
40KD
70KD
55KD
40KD
Ripk2
v5

Figure6G

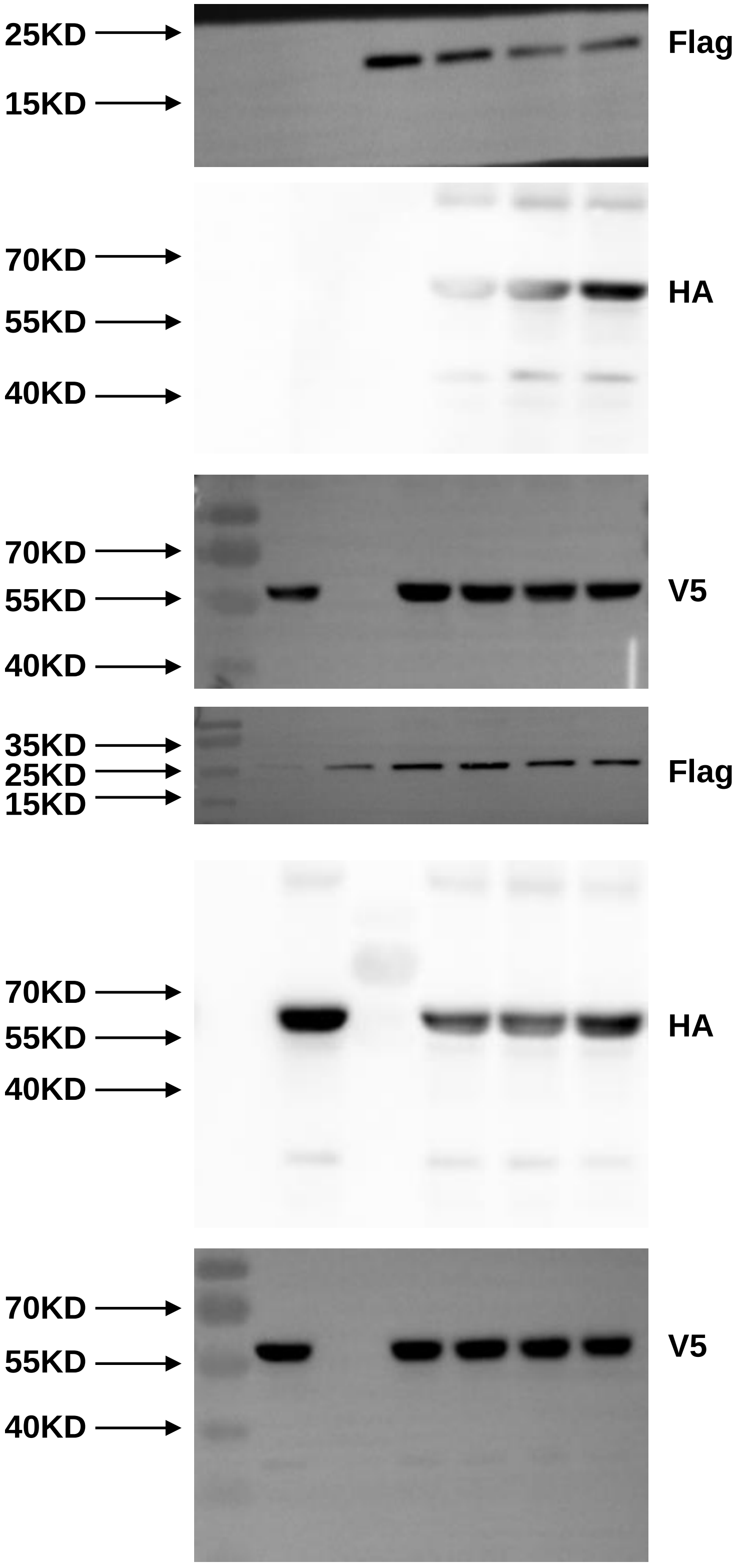
25KD
15KD
Flag
70KD
55KD
40KD
HA
70KD
55KD
40KD
V5
35KD
25KD
15KD
Flag
70KD
55KD
40KD
HA
70KD
55KD
40KD
V5

Figure S1E

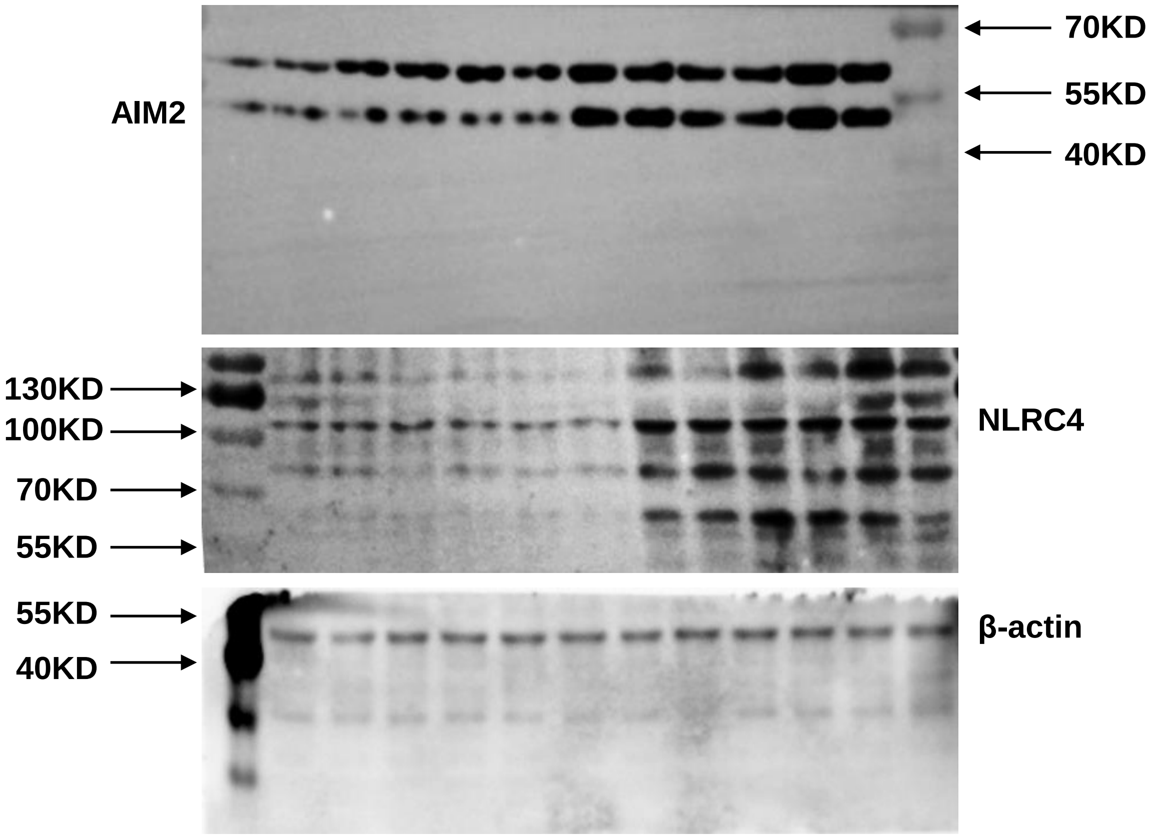
AIM2
70KD
55KD
40KD
130KD
100KD
70KD
55KD
NLRC4
55KD
40KD
β-actin

Figure S2B

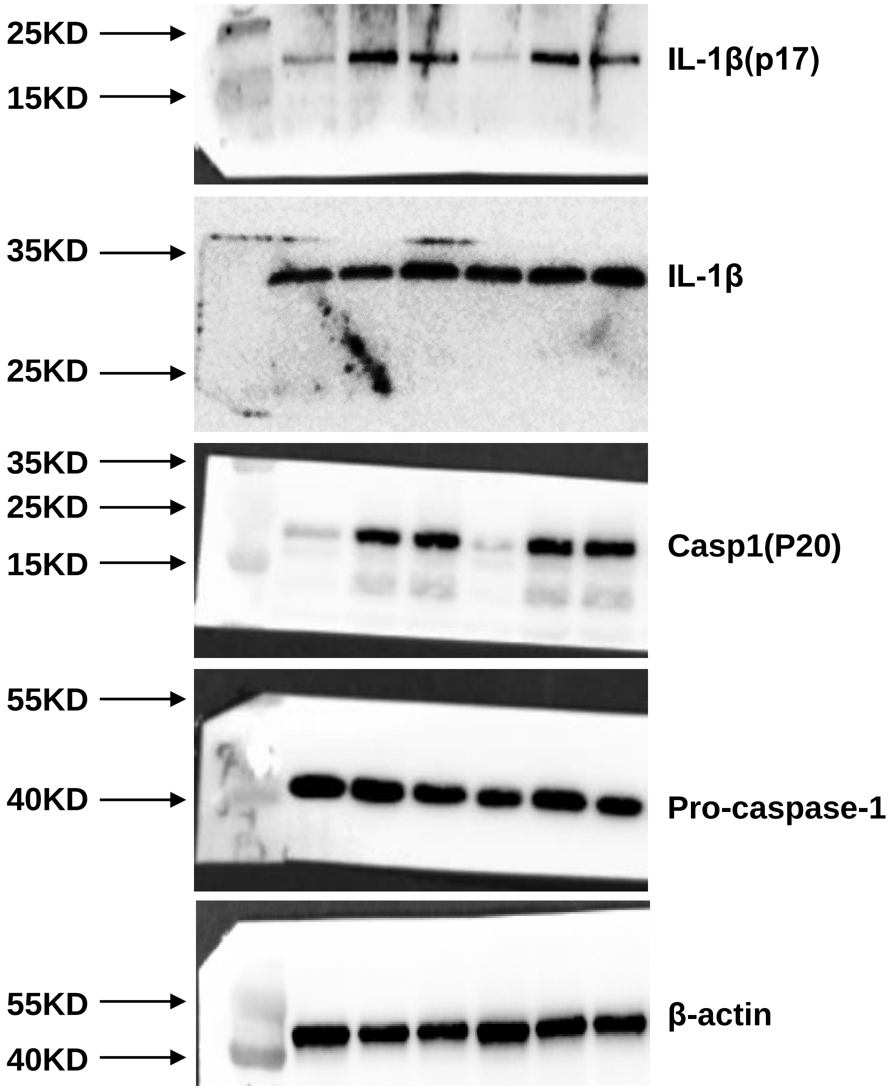
25KD
15KD
IL-1β(p17)
35KD
25KD
IL-1β
35KD
25KD
15KD
Casp1(P20)
55KD
40KD
Pro-caspase-1
55KD
40KD
β-actin

Figure S2D

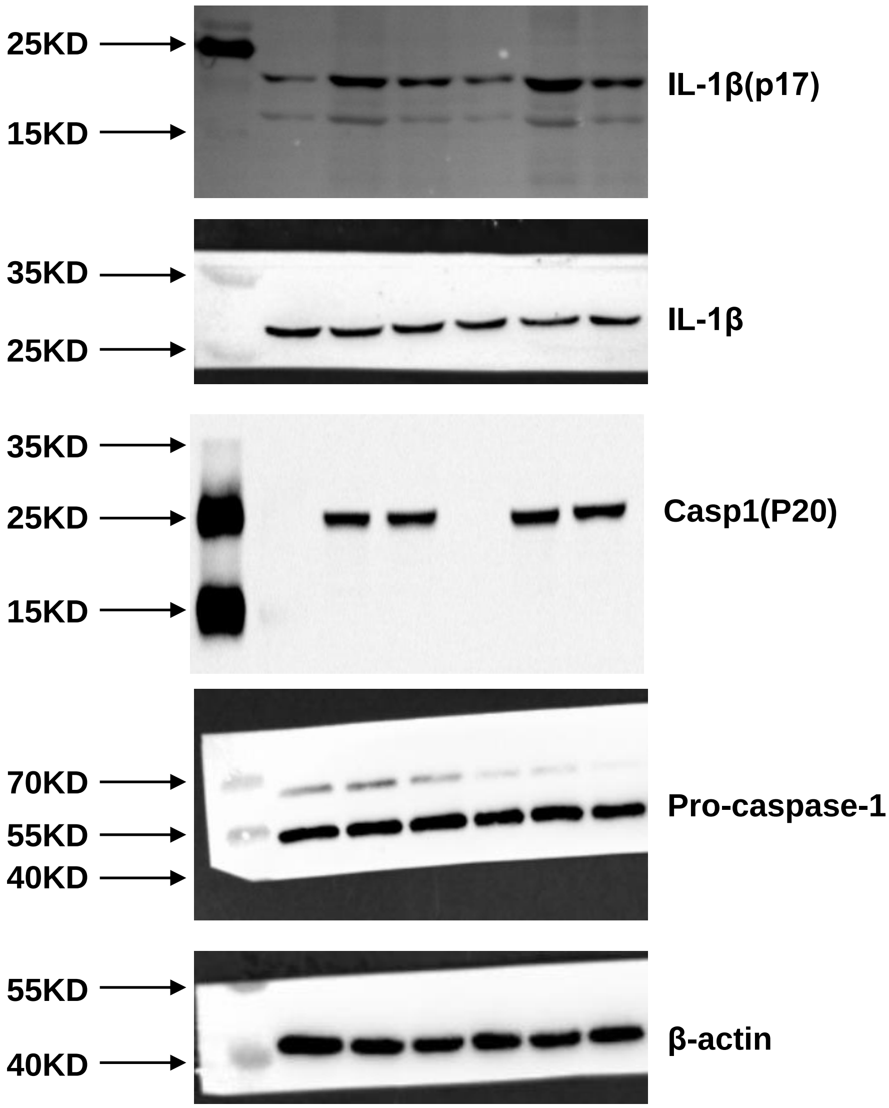
25KD
15KD
IL-1β(p17)
35KD
25KD
IL-1β
35KD
25KD
15KD
Casp1(P20)
70KD
55KD
40KD
Pro-caspase-1
55KD
40KD
β-actin

Figure S3B

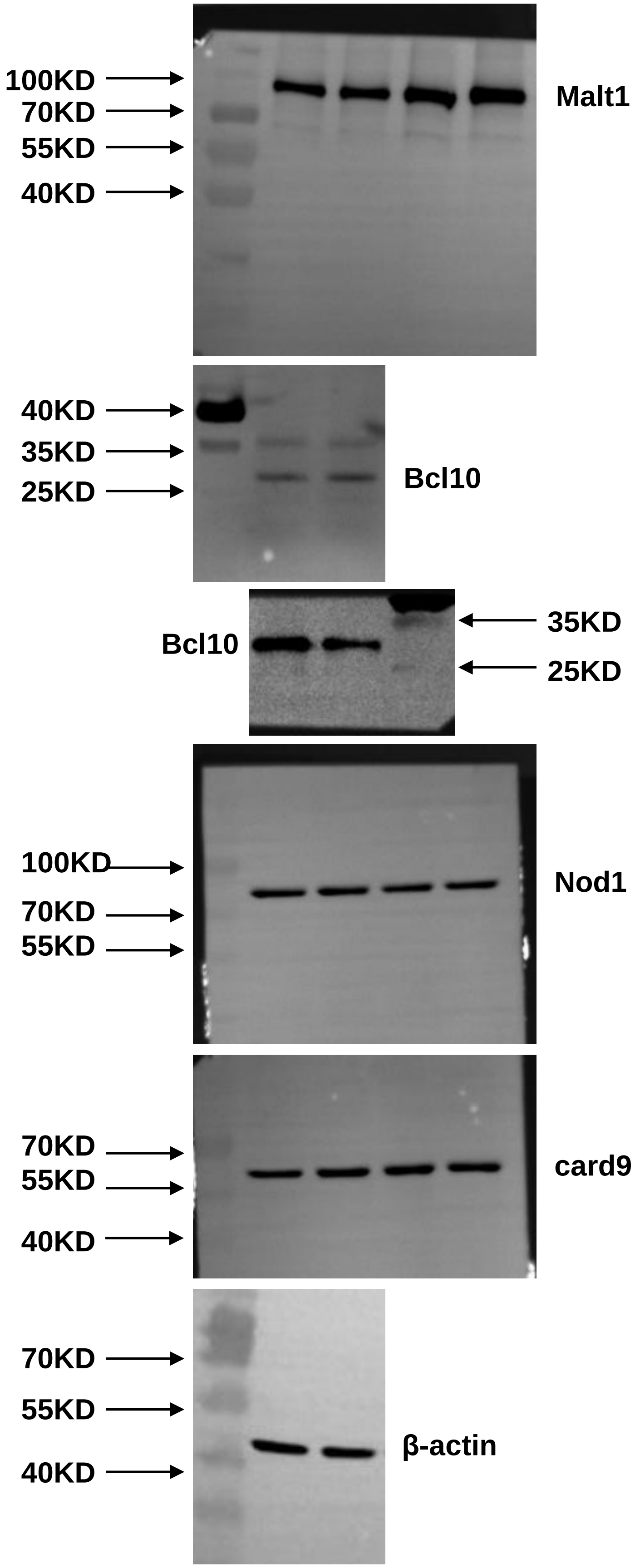
100KD
70KD
55KD
40KD
Malt1
40KD
35KD
25KD
Bcl10
Bcl10
35KD
25KD
100KD
70KD
55KD
Nod1
70KD
55KD
40KD
card9
70KD
55KD
40KD
β-actin
